# CAR T cells outperform CAR NK cells in CAR-mediated effector functions in head-to-head comparison

**DOI:** 10.1186/s40164-024-00522-6

**Published:** 2024-05-14

**Authors:** Lukas Egli, Meike Kaulfuss, Juliane Mietz, Arianna Picozzi, Els Verhoeyen, Christian Münz, Obinna Chijioke

**Affiliations:** 1 Cellular Immunotherapy, Institute of Experimental Immunology, University of Zürich, Winterthurerstrasse 190, 8057 Zurich, Switzerland; 2 International Center for Infectiology, research team Enveloped Viruses, Vectors and Innate Responses, Institut national de la Santé et de la recherche médicale, unité 1111, Unité mixte de recherche 5308, Centre national de la recherche scientifique, École Normale Supérieure de Lyon, Université Claude Bernard Lyon 1, University of Lyon, Lyon, France; 3 Université Côte d’Azur, Institut National de La Santé Et de La Recherche Médicale, Centre Méditerranéen de Médecine Moléculaire, Nice, France; 4 Viral Immunobiology, Institute of Experimental Immunology, University of Zürich, Zurich, Switzerland; 5 Institute of Medical Genetics and Pathology, University Hospital Basel, Basel, Switzerland

**Keywords:** Chimeric antigen receptor, Adoptive cell therapy, Human T cells, Human NK cells, IFN-γ, Cytotoxicity, Autologous, Allogeneic

## Abstract

**Background:**

CAR NK cells as vehicles for engineered “off-the-shelf” cellular cancer immunotherapy have attracted significant interest. Nonetheless, a comprehensive comparative assessment of the anticancer activity of CAR T cells and CAR NK cells carrying approved benchmark anti-CD19 CAR constructs is missing. Here, we report a direct head-to-head comparison of CD19-directed human T and NK cells.

**Methods:**

We generated CAR T and CAR NK cells derived from healthy donor PBMC by retroviral transduction with the same benchmark second-generation anti-CD19 CAR construct, FMC63.28z. We investigated IFN-γ secretion and direct cytotoxicity in vitro against various CD19^+^ cancer cell lines as well as in autologous versus allogeneic settings. Furthermore, we have assessed anticancer activity of CAR T and CAR NK cells in vivo using a xenograft lymphoma model in an autologous versus allogeneic setting and a leukemia model.

**Results:**

Our main findings are a drastically reduced capacity for CAR-mediated IFN-γ production and lower CAR-mediated cytotoxicity of CAR NK cells relative to CAR T cells in vitro. Consistent with these in vitro findings, we report superior anticancer activity of autologous CAR T cells compared with allogeneic CAR NK cells in vivo.

**Conclusions:**

CAR T cells had significantly higher CAR-mediated effector functions than CAR NK cells in vitro against several cancer cell lines and autologous CAR T cells outperformed allogeneic CAR NK cells both in vitro and in vivo. CAR NK cells will likely benefit from further engineering to enhance anticancer activity to ultimately fulfill the promise of an effective off-the-shelf product.

**Supplementary Information:**

The online version contains supplementary material available at 10.1186/s40164-024-00522-6.

## Background

Autologous anti-CD19 CAR T cell products have changed the treatment landscape for relapsed or refractory B cell malignancies by delivering unprecedented patient benefit, thus prompting their rapid approval in several countries [1, 2]. However, although CAR T cell therapy is potentially curative in a proportion of patients [3], there are still several limitations. These include a time-consuming production process [2], high costs [4] and severe toxicities associated with CAR T cell therapy [5, 6]. Therefore, besides engineered allogeneic CAR T cells [7], the use of allogeneic CAR NK cells is being pursued as an alternative [8], raising the prospect of more affordable and more readily available, scalable off-the-shelf products [9]. Indeed, early clinical data of treatment with partially or fully HLA-mismatched cord blood derived anti-CD19 CAR NK cells have been reported and treatment shown to be remarkably safe, with none of the patients developing neurotoxicity or graft-versus-host disease, and highly potent [10, 11]. However, while HER2 directed CAR T and CAR NK cells have been compared [12], there are no direct comparison studies to date of human anti-CD19 CAR T cells versus CAR NK cells.

Here, using a CD28 costimulatory domain-containing CAR design, as used in clinical trials of both CAR NK [10, 11] and CAR T cells [13, 14], we report significantly reduced CAR-mediated IFN-γ production and cytotoxicity after acute and chronic CAR stimulation by CAR NK cells compared with CAR T cells against various CD19 positive cancer cell lines in vitro in a head-to-head comparison. To model anticipated off-the-shelf applications, we compared autologous CAR T cells with allogeneic CAR NK cells in vitro and in vivo in a xenograft lymphoma model and found that autologous CAR T cells had superior anticancer activity.

## Methods

### Primary cells and cell lines

Primary human immune cells were purified from peripheral blood mononuclear cells (PBMC) of healthy donors by density gradient centrifugation (Ficoll Paque Premium, GE Healthcare, 17-5442-03) followed by isolation of B, T and NK cells by FACS sorting. For expansion of primary human T and NK cells, K562mbIL21 feeder cells (kindly provided by Dr. Dean Lee, Nationwide Children’s Hospital, Columbus, United States) were irradiated with 130 Gy and added once per week, starting at a 1:2 lymphocytes to feeder cell ratio on day 0 and continued with a 1:1 ratio, as described previously [15]. Expansion (complete) media for primary human NK and T cells based on RPMI1640 medium (Gibco, 52400–041) was supplemented with 10% fetal bovine serum (FBS; Merck, S0615), 1% penicillin/streptomycin (P/S; Gibco, 15140–122), 2 mM L-Glutamine (Gibco, 25030–164) and 100 IU/ml recombinant human IL-2 (PeproTech, 200–02) and replaced every 3–4 days. CAR T and CAR NK cells were expanded on feeder cells for 3–4 weeks before being used in downstream applications. Alternatively, sorted T cells were activated and expanded by CD3/CD28 co-stimulation using Dynabeads^™^ (Gibco, 11131D). After isolation, 1 × 10^6^ T cells were activated with 5 × 10^5^ beads. Expansion media based on RPMI1640 medium (Gibco, 52400–041) was supplemented with 10% fetal bovine serum (FBS; Merck, S0615), 1% penicillin/streptomycin (P/S; Gibco, 15140–122), 2 mM L-Glutamine (Gibco, 25030–164) and 50 IU/ml recombinant human IL-2 (PeproTech, 200–02) and replaced every 2–3 days. CAR T cells were expanded for 3 weeks and then used in subsequent assays.

2.5 × 10^5^ primary human B cells were plated in a 96-well flat-bottom plate and cultured with EBV supernatant (EBV B95-8; produced as previously described [16]) at an MOI of 0.1 to generate lymphoblastoid cell lines (LCL). Jurkat, NALM6, Raji, 721.221 and LCL were cultured in RPMI1640 medium supplemented with 10% FBS, 2 mM L-Glutamine and 1% P/S and passaged twice per week. Human embryonic kidney (HEK293T) cells were cultured in DMEM (Gibco, 41966–052) supplemented with 10% FBS, 2 mM L-Glutamine and 1% P/S and passaged 2–3 times per week. Cells underwent regular testing for mycoplasma using MycoSPY Master Mix (Biontex, M020-050).

### Virus production

HEK293T cells were transfected with the packaging plasmid pUMVC (Addgene #8449), the BaEVRless envelope plasmid and the second-generation anti-CD19 CAR vector plasmid MSGV FMC63.28z, containing a CD28 costimulatory domain (kindly provided by James N. Kochenderfer, NCI, Bethesda, USA), at a ratio of 1:1:4 using polyethyleneimine (PEI) at DNA:PEI ratio of 1:6. Supernatant was collected 48 h after transfection and either used directly or stored at −80 °C until further use.

### Retroviral transduction

On day 3 after isolation, 0.5–1 × 10^6^ NK or T cells were resuspended in 0.5–1 ml of viral supernatant and transduced in a 24-well plate by spinfection (1.5 h, 1500xg, 32 °C) and in the presence of 0.8 µg/ml polybrene (EMD Millipore, TR-1003-G). Viral supernatant was replaced by fresh complete medium immediately after spinfection. CAR expression was assessed by flow cytometry 3 days after transduction.

### Editing of target cells

CD19 negative 721.221 cells were generated by CRISPR/Cas9-mediated knockout (KO) of CD19. The crRNA (CGAGGAACCTCTAGTGGTGA) was designed in-house using CRISPOR [17] and purchased from Integrated DNA Technologies (IDT) as well as the tracrRNA (IDT, 1072533), and Cas9 (*Streptococcus pyogenes*) protein (IDT, 1081058). The formation of Cas9 ribonucleoproteins (RNPs) was performed as described by Roth et al [18]. 2.5 × 10^6^ LCL 721.221 cells were electroporated per 100 µl Nucleocuvette^™^ with RNPs using the P3 primary cell 4D-Nucleofector^™^ X kit L, 12 reactions (Lonza, V4XP-3012) and pulsed with code DN-100. FACS sorting of CD19 negative cells was performed using a FACSAria III cell sorter (BD), nozzle size 130 µm. CD19 KO efficiency was confirmed and regularly checked by flow cytometry.

### Flow cytometry

Surface markers cells were stained for 20 min at 4 °C in PBS, washed, and in case of viral transduction fixed with 1% paraformaldehyde-containing PBS. For intracellular markers, cells were fixed with the BD Pharmingen^™^ Transcription factor buffer set (BD, 562574) and then stained for 1 h at 4 °C. Dead cells were detected using Zombie Aqua^™^ (Biolegend, 423101), Zombie NIR (Biolegend, 423105) or for functional assays with TO-PRO^™^-3 Iodide (Invitrogen, T3605). For cells transduced with the MSGV FMC63.28z, CAR expression was assessed using PE-labeled recombinant human CD19 (Acro Biosystems, CD9-HP2H3) according to the manufacturer’s instructions. Samples were acquired on a BD LSRFortessa^™^ flow cytometer (BD) or Cytek Aurora (Cytek). Flow data was analyzed using FlowJo software (Tree Star). The following antibodies were used (fluorochrome, clone, manufacturer, catalogue number; if not stated otherwise all antibodies were anti-human): CD103 (BV711, Ber-ACT8, Biolegend, 350222), CD119 (PE, GIR-208, Biolegend, 308606), CD158a,h (APC, EB6B, Beckman Coulter, PNA22332), CD158b1/b2,j (APC, GL183, Beckman Coulter, PNA22333), CD158e1/2 (APC, DX9, R&D Systems, FAB1225A), CD159a (PE-Cy5, S19004C, Biolegend, 375111), CD16 (BUV, 3G8, BD, 612786), CD19 (BUV661, HIB19, BD, 741604), CD19 (BV785, HIB19, Biolegend, 302240), CD2 (BV605, RPA-2.10, Biolegend, 300,224), CD3 (BV785, OKT3, Biolegend, 317329), CD3 (BUV496, UCHT1, BD, 612941), CD3 (FITC, UCHT1, Biolegend, 300406), CD39 (PE-Fire 810, A1, Biolegend, 328245), CD4 (BUV496, SK3, BD, 564652), CD4 (APC, OKT4, Biolegend, 317416), CD45 (BUV395, HI30, BD, 563792), CD45 (Pacific Blue, HI30, Biolegend, 304029), CD45RA (FITC, HI100, Biolegend, 304106), CD49a (FITC, TS2/7, Biolegend, 328308), CD56 (PE-Cy7, NCAM16, BD, 335826), CD57 (PE-Dazzle594, HNK-1, Biolegend, 359619), CD62L (Pacific Blue, DREG-56, Biolegend, 304825), CD69 (BUV805, FN50, BD, 748763), CD8 (BUV563, RPA-T8, BD, 612914), CD8 (BV650, SK1, Biolegend, 344730), EOMES (PerCP-eFluor710, eBioscience, 46-4877-42), Granzyme B (Alexa Fluor 700, GB11, BD, 560213), HLA-DR (PE-Fire 640, L243, Biolegend, 307676), Ki67 (BV605/BV711, Ki-67, Biolegend, 350522/350516), PD1 (BUV737, EH12.1, BD, 612792), Perforin (BV421, dG9, Biolegend, 308122), Tbet (BV650, O4-46, BD, 564142), TCF1 (PE, 7F11A10, Biolegend, 655207), Tim3 (BV650, 7D3, BD, 565565), Tim3 (APC-Cy7, F83-2E2, Biolegend, 345025), TOX (APC, REA-473, Miltenyi, 130-118-335).

### Functional in vitro* assays*

For cytotoxicity assays, 721.221, NALM6, Raji and LCL were labeled with PKH26 red fluorescent cell linker (Sigma, MINI26-1KT). Labeled target cells and primary cells were cocultured for 4 h at the indicated effector to target (E:T) ratio. Right before acquisition, TO-PRO^™^-3 Iodide, was added to the sample [19]. Specific lysis was calculated as % dead labeled target cells—% spontaneous lysis of labeled target cells. For restimulation, effector cells were cocultured in fresh medium with or without IL-2 with unlabeled target cells for 24 h at an E:T ratio of 5:1. Medium was then replaced with medium without IL-2 and labeled target cell were added. For long-term coculture, 1 × 10^5^ effector cells were seeded in a 96-well plate and 2 × 10^4^ target cells added. At the indicated time points, half of the medium was exchanged and 2 × 10^4^ unlabeled target cells and 100 IU/ml IL-2 added. On the last day, medium was replaced completely with medium without IL-2 and 2 × 10^4^ labeled target cell were added.

After coculture, the concentration of secreted IFN-γ was measured according to the manufacturer’s protocol using the Human IFN-gamma DuoSet ELISA (R&D, DY285B-05). Alternatively, effector cells were stimulated with 50 ng/ml phorbol 12-myristate 13-acetate (PMA, Sigma, P1585) and 0.65 µM ionomycin (Biolegend, 420701) for 4 h and IFN-γ measured by ELISA. Plates were acquired using an ELISA reader (infinite M200Pro, Tecan) and analyzed with the i-control 2.0 software (Tecan).

CAR-mediated effector functions (cytotoxicity and IFN-γ production) were calculated by subtracting values of untransduced cells from the transduced cells of the same donor.

### In vivo experiments

NSG (NOD.Cg-Prkdc < scid > Il2rg < tm1Wjl > /SzJ (#005557)) mice were purchased from The Jackson Laboratory and bred and housed under specific pathogen-free conditions at the Laboratory Animal Services Center (LASC) Zurich, University of Zurich. All animal experiments were performed according to an approved license by the veterinary office of the canton of Zurich, Switzerland (ZH049/20).

Subcutaneous LCL model: LCL tumors were injected subcutaneously into the left flank under isoflurane narcosis. 2 × 10^6^ tumor cells were resuspended in PBS and right before injection mixed at a 1:1 V/V ratio with Corning^®^ Matrigel^®^ Growth Factor Reduced (GFR) Basement Membrane Matrix (Corning, 354230).

Three days after tumor injection, 4 × 10^6^ or 10 × 10^6^ effector cells were adoptively transferred by tail vein injection and mice injected intraperitoneally (i.p.) with 1 × 10^5^ IU IL-2 every second day. Tumor size was monitored by calipering (3x/week or daily). Tumor volume was approximated with the following formula:

$$V=\frac{{{\text{L}}}^{2}\times W}{0.52}$$

Systemic NALM6 model: 5 × 10^5^ luciferase-expressing NALM6 cells (kindly provided by Dr. Chiara Magnani, University Hospital Zurich, Switzerland) were injected intravenously. Four days after tumor inoculation, effector cells were adoptively transferred by tail vein injection and mice injected intraperitoneally (i.p.) with 1 × 10^5^ IU IL-2 every second day. Tumor growth was assessed weekly by measuring bioluminescence after intraperitoneal injection of luciferin (VivoGlo^™^ Luciferin, Promega, P1043). Measurements were performed using the IVIS Lumina (PerkinElmer) and data analyzed with the Living Image Software (PerkinElmer).

General health was monitored by weighing and health parameter scoring (3x/week or daily, according to animal license). Persistence of adoptively transferred cells was monitored by tail vein bleeding. Blood was treated with ammonium chloride potassium (ACK) lysis buffer (Gibco), washed with PBS, stained and analyzed by flow cytometry.

At sacrifice, blood was collected by heart puncture and treated with ACK lysis buffer. Spleens were meshed through a 70 µm cell strainer and lymphocytes isolated by density gradient centrifugation. For isolation of tumor infiltrating lymphocytes, skin covering the tumor was removed and tumors cut into pieces with scissors, digested in DMEM with 2% heat-inactivated FBS and supplemented with 1 mg/mL collagenase IV (Roche, 11088866001), 40 µg/mL DNase I (Roche, 4716728001) and 1.2 mM CaCl_2_ for 45 min at 37 °C and 200 rpm, meshed through a 70 μm cell strainer and washed twice with PBS. All single cell suspensions were then stained and analyzed by flow cytometry.

### Statistics

Statistical analysis was performed using Prism 10 Software (GraphPad Software, Inc). p values lower than 0.05 were considered significant. The statistical tests used as well as the number of biological replicates are specified in the figure legends.

## Results

### Generation and expansion of anti-CD19 human CAR T and CAR NK cells

**Fig. 1:**
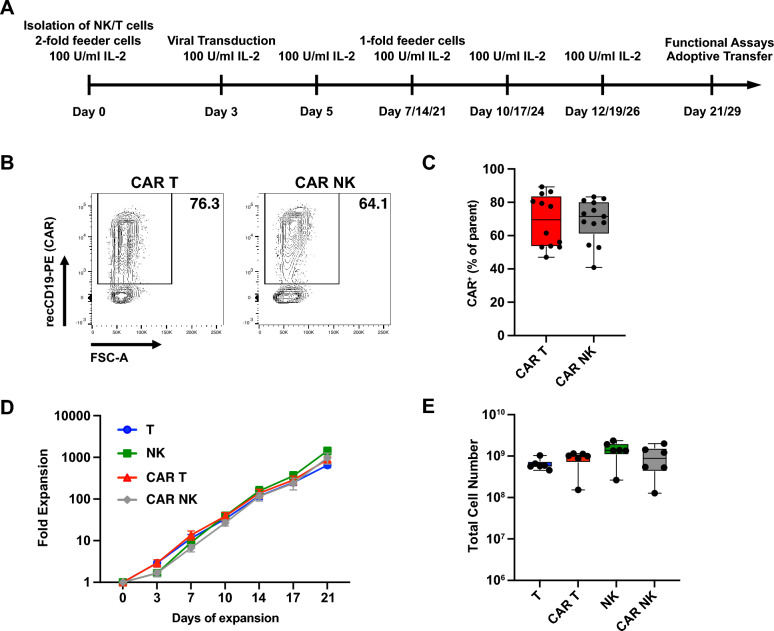
Generation and expansion of CAR T and CAR NK cells. **A** Timeline of the generation and expansion of immune effector cells. (B, C) PBMC-derived primary human T and NK were transduced with a retroviral vector encoding an anti-CD19 CAR (FMC63.28z). Three days after transduction, CAR expression was measured by flow cytometry after staining with PE-labelled recombinant CD19. **B** Representative contour plot showing CAR expression on CAR T cells (left) and CAR NK cells (right). **C** Summary of transduction rates of T and NK cells. **D**, **E** CAR and untransduced cells were expanded for three weeks with weekly stimulation with K562mbIL21 feeder cells with addition of 100 U/ml IL-2. Logarithmic expansion over 3 weeks (**D**) and total expansion on day 21 (**E**). Data are from 5 independent experiments with n = 12 donors (**C**) and from 2 independent experiments with n = 6 donors (**D**, **E**). Significance by paired t-test (C) and RM one-way ANOVA with Tukey’s multiple comparisons (**E**). Symbols in (**C**) and (**E**) represent individual donors. Data shown as box and whiskers display the median as a line within the box; whiskers are shown from minimum to maximum data point

After isolation by FACS sorting (Fig. S1A) from healthy donor PBMCs, both human T and human NK cells were expanded using a feeder cell line (K562 cells expressing membrane-bound IL-21 [20]) and IL-2 supplementation, as has been reported previously for expansion of NK cells in clinical trials [10, 11, 21, 22] (Fig. 1A). Following an initial expansion period of three days, both T and NK cells were efficiently transduced using a retroviral vector pseudotyped with a baboon retroviral envelope glycoprotein [23], resulting in high level expression in both cell types of a second-generation anti-CD19 CAR (FMC63.28z [24]) with a mean of 64.5% CAR T cells and a mean of 70.35% CAR NK cells (Fig. 1B, C). Over the course of three weeks, both transduced and untransduced human T and NK cells could be expanded over 1000-fold on average, with high lineage purity maintained (Fig. S1B) and without significant expansion differences between T and NK cells or transduced and untransduced cells (Fig. 1D, E). Taken together, this protocol allowed for clinically relevant ex vivo expansion of human T and NK cells and high transduction efficiencies, thereby obviating the need for sorting of CAR cells.

### Phenotype of expanded CAR T and CAR NK cells

**Fig. 2:**
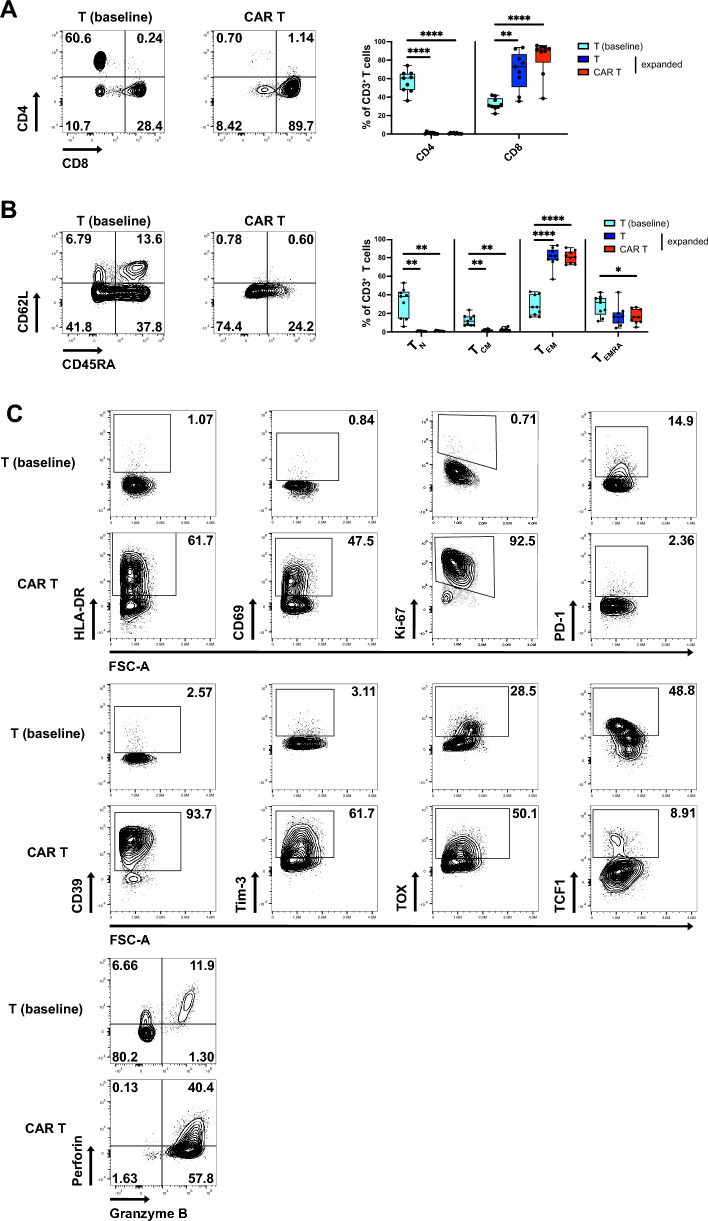

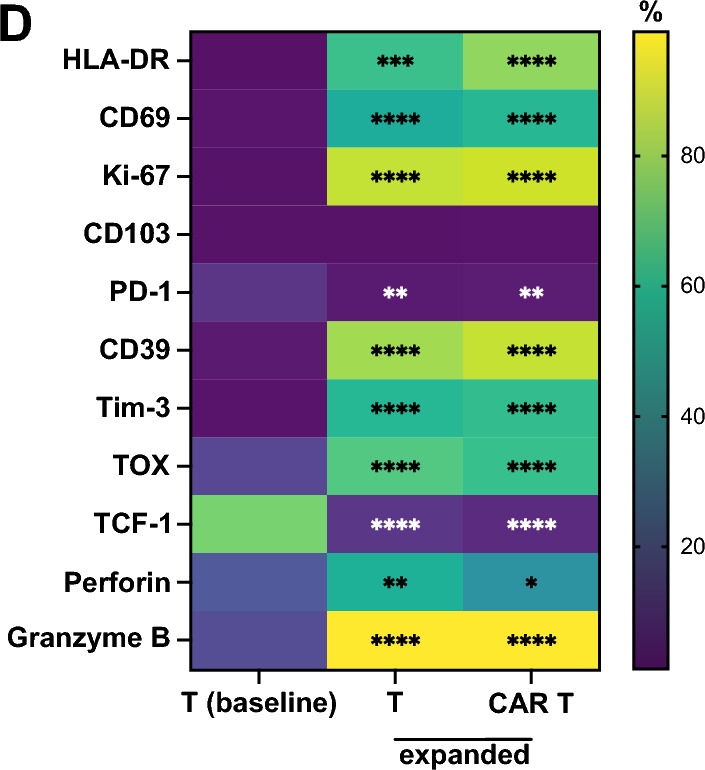
Phenotype of expanded CAR T and untransduced T cells CAR T cells or untransduced T cells were expanded for 3 weeks and phenotype analyzed by flow cytometry. **A** Flow cytometric analysis of CD4 and CD8 expression and percentages of CD4^+^ and CD8^+^ T cells within freshly isolated CD3^+^ T cells (baseline), expanded untransduced T cells and expanded CAR T cells. **B** Flow cytometric analysis of T cell differentiation and percentages of naïve (T_N_, CD45RA^+^CD62L^+^), central memory (T_CM_, CD45RA^−^CD62L^+^), effector memory (T_EM_, CD45RA^−^CD62L^−^) and T_EMRA_ (CD45RA^+^CD62L^−^) cells within T cell populations. (**C**) Representative contour plots showing marker expression in freshly isolated CD3^+^ T cells and expanded CAR T cells. (**D**) Heat map showing differences in marker expression in CD3^+^ T cells freshly isolated from PBMCs vs. expanded untransduced T cells and freshly isolated CD3^+^ T cells vs. expanded CAR T cells. Data are from 3 independent experiments with n = 6–9 donors. Significance by RM one-way ANOVA with Tukey’s multiple comparisons or 2way ANOVA with Tukey’s multiple comparisons as appropriate; *p < 0.05, **p < 0.01, ***p < 0.001, ****p < 0.0001. Symbols in (**A**) and (**B**) represent individual donors. Data shown as box and whiskers display the median as a line within the box; whiskers are shown from minimum to maximum data point

The expansion of both CAR T cells and untransduced T cells led to the preferential expansion of CD8^+^ T cells (Fig. 2A**).** The majority of expanded T cells displayed an effector memory (T_EM_)-like phenotype (CD45RA^−^CD62L^−^) with a minor fraction of T_EMRA_ (CD45RA^+^CD62L^−^) cells (Fig. 2B). Post-expansion, both untransduced T cells and CAR T cells exhibited increased expression of the activation and proliferation markers HLA-DR, CD69 and Ki-67 in comparison to baseline T cells as well as upregulation of the exhaustion markers CD39 and Tim-3, while PD-1 was reduced (Fig. 2C, D and Fig. S2B). The cytolytic effector molecules perforin and especially granzyme B were upregulated as was the exhaustion-associated transcriptional regulator TOX [25] (Fig. 2C, D and Fig. S2B), while TCF-1, marking stemness and progenitor populations [26], was downregulated (Fig. 2C, D and Fig. S2B). We did not detect major phenotypic differences between untransduced and CAR T cells after expansion, except for slightly upregulated HLA-DR and lower perforin expression in CAR T cells (Fig. S2A, B). Expression of NK cell markers (KIR, CD56) was increased in expanded untransduced and CAR T cells (Fig. S2C). We also generated CAR T cells using a protocol based on CD3/CD28 costimulation, as this is more commonly used for clinical CAR T cell products [27, 28] (Fig. S3A). We observed similar transduction rates (Fig. S3B), but a higher fraction of CD4^+^ T cells (Fig. S3C) and relative higher abundance of naïve and central memory-like phenotypes (Fig. S3D). Furthermore, these CAR T cells exhibited a less activated/exhausted-like phenotype relative to CAR T cells expanded with feeder cells (Fig. S3E). However, the potency of the feeder-based CAR T cell product was comparable to that of the CD3/CD28-based CAR T cell product, as evidenced by similar levels of cytotoxicity (Fig. S3F). Together, expanded CAR T cells exhibited a highly activated effector-like phenotype with distinct expression of exhaustion markers.

**Fig. 3:**
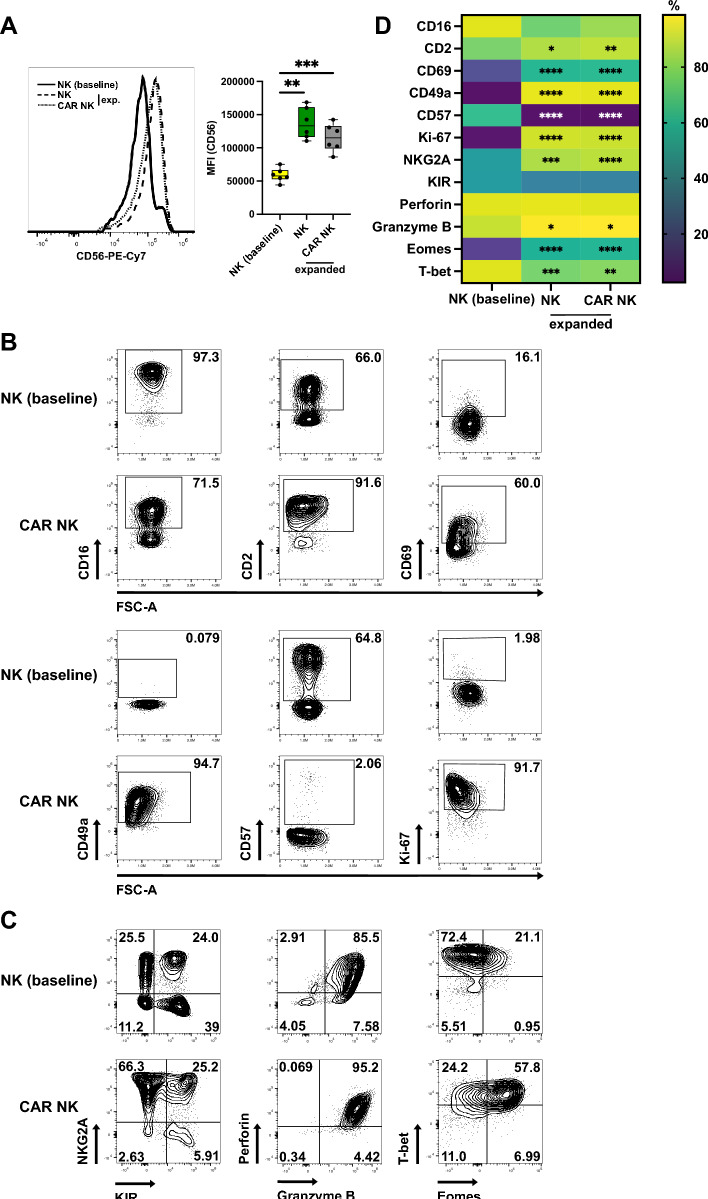
Phenotype of expanded CAR NK and untransduced NK cells. CAR NK cells or untransduced NK cells were expanded for 3 weeks and phenotype analyzed by flow cytometry. **A** Representative histograms of CD56 expression (left) and average CD56 intensity (MFI) on freshly isolated CD3^−^CD56^+^ NK cells (baseline) and expanded NK populations. (B, C) Representative contour plots showing expression of markers of NK cell differentiation, residency and proliferation (**B**), NKG2A, KIR, cytolytic effector molecules as well as transcription factors T-bet and Eomes (**C**) on freshly isolated NK cells and expanded CAR NK cells. **D** Heat map showing differences in marker expression in NK T cells freshly isolated from PBMCs vs. expanded untransduced NK cells and freshly isolated NK cells vs. expanded CAR NK cells. Data are from 3 independent experiments with n = 5–9 donors. Significance by RM one-way ANOVA with Tukey’s multiple comparisons or one-way ANOVA with Tukey’s multiple comparisons as appropriate; *p < 0.05, **p < 0.01, ***p < 0.001, ****p < 0.0001. Symbols in (**A**) represent individual donors. Data shown as box and whiskers display the median as a line within the box; whiskers are shown from minimum to maximum data point

Expanded NK cells showed higher intensity of CD56 compared with freshly isolated NK cells, which is not unexpected as CD56 has also been described as an NK cell activation marker [29] (Fig. 3A). CD16 and CD2, both being critical for antibody-dependent cell-mediated cytotoxicity (ADCC) [30, 31], showed opposite changes in expression post-expansion. Whereas CD16 was slightly downregulated, CD2 was expressed at higher frequencies on NK and CAR NK cells after expansion (Fig. 3B, D and Fig. S4B). The activation and residency marker CD69 was upregulated as was CD49a, usually a marker for tissue residency. Interestingly, expansion resulted in reduced frequencies of CD57^+^ NK cells, likely due to depletion of terminally differentiated NK cells within the highly proliferative (Ki-67^+^) NK cell population (Fig. 3B, D and Fig. S4B). The inhibitory receptor NKG2A was markedly upregulated while expression of killer cell immunoglobulin-like receptors (KIRs) was slightly but non-significantly decreased (Fig. 3C, D and Fig. S4B). The cytolytic effector molecules perforin and granzyme B were co-expressed in the majority of freshly isolated NK cells and both remained high or even had increased expression after expansion (Fig. 3C, D and Fig. S4B). Almost all freshly isolated NK cells expressed T-bet and most co-expressed Eomes, both of which are important transcription factors for NK cell maturation and effector functions [32]. Over the course of expansion, T-bet was downregulated while Eomes expression was increased (Fig. 3C, D and Fig. S4B). Similar to expanded T cells, we did not observe major phenotypic differences between untransduced NK cells and CAR NK cells post-expansion (Fig. S4A, B). Thus, expanded CAR NK cells acquired a phenotype that resembled a not fully matured [33] but activated and cytotoxic NK cell state, a phenotype driven by expansion but not transduction itself.

### In vitro functionality of expanded CAR T and CAR NK cells

**Fig. 4:**
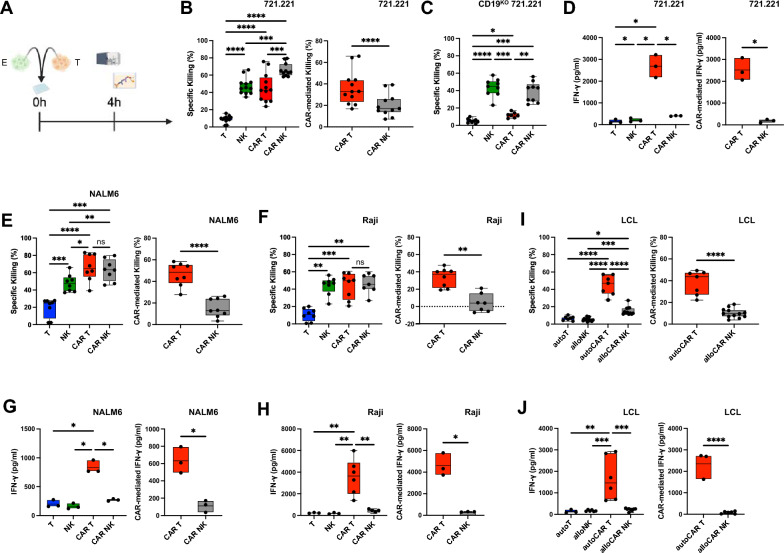
In vitro functionality of CAR T and CAR NK cells. Expanded cells were cocultured with different cancer cell lines at an effector to target (E:T) ratio of 5:1 for 4 h. Specific killing was assessed by flow cytometry using a membrane-impermeable DNA stain (TO-PRO-3) and PKH26 membrane-labeled target cells and IFN-γ production was measured by ELISA. **A** Schematic of the timeline. **B**, **C** Specific (left) and CAR-mediated (right) killing of CD19^+^ 721.221 (**B**) and CD19^KO^ 721.221 cells (**C**) by anti-CD19 CAR T, CAR NK cells or untransduced controls. **D** Overall (left) and CAR-mediated (right) IFN-γ production by anti-CD19 CAR T, CAR NK cells and untransduced controls after coculture with CD19^+^ 721.221 cells. (E, F) Specific (left) and CAR-mediated (right) killing of NALM6 (**E**) or Raji (**F**) cells by anti-CD19 CAR T, CAR NK cells and untransduced controls. **G**, **H** Overall (left) and CAR-mediated (right) IFN-γ production by anti-CD19 CAR T, CAR NK cells and untransduced controls after coculture with NALM6 (**G**) or Raji (**H**) cells. **I** Specific (left) and CAR-mediated (right) killing of LCL by autologous anti-CD19 CAR T cells, allogeneic anti-CD19 CAR NK cells and untransduced controls. **J** Overall (left) and CAR-mediated (right) IFN-γ production by autologous anti-CD19 CAR T cells, allogeneic anti-CD19 CAR NK cells and untransduced controls after coculture with LCL target cells. Data are from 1 to 3 independent experiments with n = 3–12 donors. Significance by mixed-effects analysis, RM one-way ANOVA with Tukey’s multiple comparisons, ordinary one-way ANOVA with Tukey’s multiple comparisons, two-tailed paired t-test or two-tailed unpaired t-test as appropriate; *p < 0.05, **p < 0.01, ***p < 0.001, ****p < 0.0001. Symbols represent individual donors. Data shown as box and whiskers display the median as a line within the box; whiskers are shown from minimum to maximum data point

Next, we investigated the functionality of CAR T and CAR NK cells, including untransduced expanded T and NK cells, by short-term coculture with a variety of CD19 positive cancer cell lines, assessing direct cytotoxicity as well as IFN-γ production (Fig. 4A). As expected, the HLA class I-deficient lymphoblastoid 721.221 cell line was killed efficiently by NK cells but not by T cells. For both effector cell types, redirection with the anti-CD19 CAR FMC63.28z significantly increased killing of CD19^+^ 721.221 with higher cytolytic activity in CAR NK cells than in CAR T cells, however CAR-mediated killing was significantly more pronounced in CAR T cells (Fig. 4B, Fig. S5B). CD19^KO^ 721.221 target cells were not killed at higher rates by CAR NK cells than by untransduced NK cells, confirming specificity of the CAR (Fig. 4C). A small but significant increase in killing of CD19^KO^ 721.221 cells by CAR T cells compared with untransduced T cells (Fig. 4C) was most likely due to some remaining CD19^+^ cells within the CD19^KO^ population (Fig. S5A). Untransduced NK cells produced significantly more IFN-γ compared with untransduced T cells after coculture with 721.221 cells, however, at a range well below 500 pg/ml. In contrast, CAR T cells exhibited log-fold higher IFN-γ production in comparison to both untransduced effector cells and a more than sixfold increase relative to CAR NK cells (2687.8 pg/ml vs. 405.7 pg/ml; Fig. 4D), demonstrating that CAR-mediated IFN-γ production was substantially higher in CAR T cells compared to CAR NK cells.

We observed a similar pattern of enhanced CAR-mediated cytotoxicity and IFN-γ production by CAR T cells relative to CAR NK cells for two additional cancer cell lines, NALM6, an acute lymphoblastic leukemia cell line, and Raji, a Burkitt lymphoma cell line (Fig. 4E–H). Untransduced NK cells, being innate lymphocytes, killed NALM6 cells more efficiently compared with untransduced T cells, but CAR incorporation had a significantly more pronounced effect on increasing cytotoxicity in CAR T cells than in CAR NK cells relative to untransduced counterparts (Fig. 4E, Fig. S5C). Similarly, while killing of Raji cells was higher with untransduced NK cells compared to untransduced T cells, CAR T cells exhibited significantly higher CAR-mediated killing of these target cells, an effect almost absent in CAR NK cells (Fig. 4F, Fig. S5D). Untransduced T and NK cells produced IFN-γ at comparatively low levels after coculture with either target cell line (Fig. 4G, H). As with 721.221 targets, redirection with the CAR led to drastically higher IFN-γ production by CAR T cells than by CAR NK cells upon coculture with both NALM6 and Raji cells (Fig. 4G, H).

There is considerable interest in the use of allogeneic CAR NK cells as off-the-shelf products, whereas CAR T cells have so far only been approved as autologous products. In a corresponding translational approach, we generated autologous CAR T cells and allogeneic CAR NK cells and tested effector responses after CAR stimulation by short-term coculture. CD19 positive target cell lines, being either autologous or allogeneic to their respective effector cells, were produced in-house by transformation of primary human B cells with Epstein-Barr virus (EBV), yielding lymphoblastoid cell lines (LCL). While killing was low for both untransduced T and NK cells, autologous CAR T cells killed their targets much more efficiently than allogeneic CAR NK cells (42.9% vs. 15.2%) and CAR-mediated killing was significantly higher in autologous CAR T cells than in allogeneic CAR NK cells (Fig. 4I, Fig. S5E). Again, we observed a similar pattern for IFN-γ with much higher production by autologous CAR T cells compared with allogeneic CAR NK cells (2484.9 pg/ml vs. 246.7 pg/ml; Fig. 4J), confirming our previous findings. Thus, CAR NK cells were less efficient in producing IFN-γ in cocultures with various target cells, including freshly generated LCL. To test the maximum capacity for IFN-γ production, we stimulated effector cells with PMA and ionomycin. IFN-γ was produced at sixfold lower levels in both untransduced NK cells (795.5 pg/ml) and CAR NK cells (1143.2 pg/ml) in comparison to untransduced T cells (4998.7 pg/ml) and CAR T cells (6870.7 pg/ml), being slightly higher in both CAR T and CAR NK cells relative to the untransduced controls (Fig. S6A). IFN-γ itself did not affect the expansion of CAR NK cells, as neither blocking nor adding IFN-γ had an effect (Fig. S6B), although IFN-γ-R1 (CD119) was present on CAR NK cells, albeit at low but detectable levels (Fig. S6C).

Together, NK cells exhibited higher intrinsic cytolytic activity against various cancer cell lines compared with T cells, in line with their well-established innate capacity to eradicate tumor cells [34]. Consequently, the overall killing capacity of CAR NK cells was equal or better compared to CAR T cells against most cancer cell lines. In contrast, CAR-mediated killing by CAR T cells was significantly higher compared to CAR NK cells across target cells, including autologous CAR T cells, which were superior to allogeneic CAR NK cells in terms of killing freshly generated LCL tumor cells. Remarkably, CAR T cells produced far more IFN-γ than CAR NK cells in all target cell cocultures tested and also upon PMA and ionomycin stimulation, suggesting an overall reduced ability of CAR NK cells for IFN-γ production relative to CAR T cells.

### In vitro functionality after long-term chronic and repetitive CAR stimulation

**Fig. 5:**
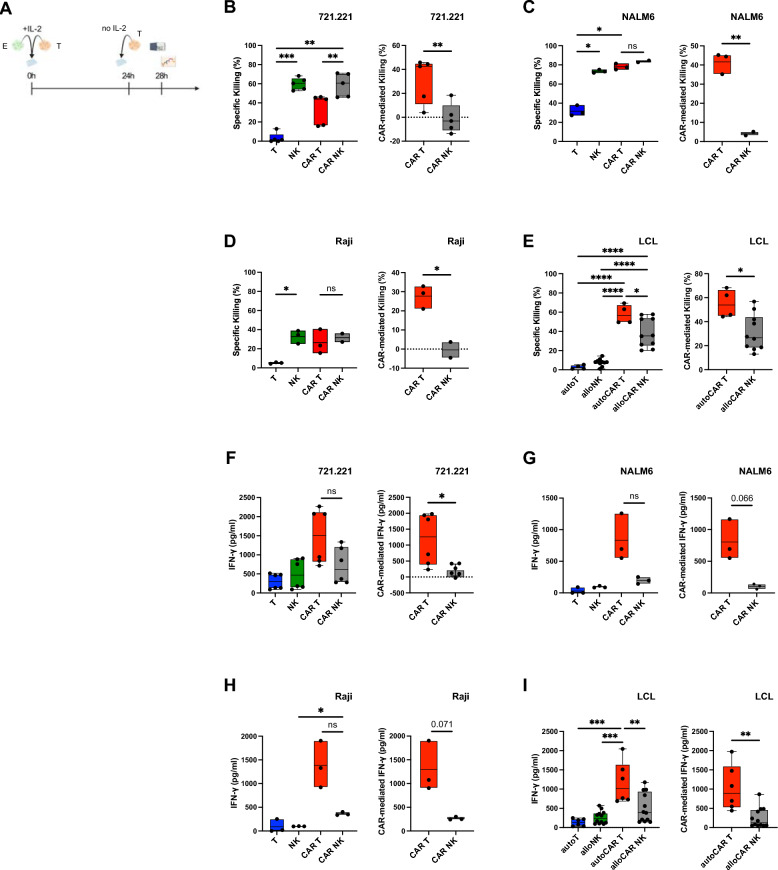
In vitro functionality of CAR T and CAR NK cells after restimulation. Expanded cells were cocultured for 24 h with the indicated cancer cell lines in the presence of 100 U/ml IL-2 and restimulated once. Specific killing after the final 4-h target cell restimulation without IL-2 was assessed by flow cytometry using a membrane-impermeable DNA stain (TO-PRO-3) and PKH26 membrane-labeled target cells. IFN-γ production was measured by ELISA. **A** Schematic of the timeline for single restimulation experiments (*E* effector cells. *T* target cells). **B**–**E** Specific (left) and CAR-mediated (right) killing after single restimulation of 721.221 cells (**B**), NALM6 cells (**C**) or Raji cells (**D**) by anti-CD19 CAR T, CAR NK cells and untransduced controls and of LCL (**E**) by autologous anti-CD19 CAR T cells, allogeneic anti-CD19 CAR NK cells and untransduced controls. (F-I) Overall (left) and CAR-mediated (right) IFN-γ production after single restimulation with 721.221 cells (**F**), NALM6 cells (**G**) or Raji cells (**H**) by anti-CD19 CAR T, CAR NK cells and untransduced controls and with LCL (**I**) by autologous anti-CD19 CAR T cells, allogeneic anti-CD19 CAR NK cells and untransduced controls. Data are from 1 to 2 independent experiments with n = 2–10 donors. Significance by mixed-effects analysis, RM one-way ANOVA with Tukey’s multiple comparisons, ordinary one-way ANOVA with Tukey’s multiple comparisons, two-tailed paired t-test or two-tailed unpaired t-test as appropriate; *p < 0.05, **p < 0.01, ***p < 0.001, ****p < 0.0001. Symbols represent individual donors. Data shown as box and whiskers display the median as a line within the box; whiskers are shown from minimum to maximum data point

We next performed long-term single restimulation assays with IL-2 preactivated effector cells (Fig. 5A). Both untransduced NK cells and CAR NK cells were highly dependent on IL-2 preactivation, as killing of the NK cell sensitive 721.221 cell line was greatly reduced and virtually absent in LCL without preactivation (Fig. S6D). We observed a similar effect for CAR T cells, especially with LCL as targets, but to a much smaller extent (Fig. S6D). Preactivated untransduced NK cells killed 721.221, NALM6 and Raji cells at a high level after restimulation, while no further increase in killing was obtained with CAR NK cells, thus CAR-mediated killing was minimal in CAR NK cells (Fig. 5B–D). In contrast, cytolytic activity against allogeneic LCLs was significantly higher in CAR NK cells relative to untransduced NK cells (Fig. 5E). CAR T cells efficiently killed target cells, albeit with lower cytolytic activity against 721.221 cells, but similar for NALM6 and Raji and even higher for LCL (Fig. 5B–E). Notably, CAR-mediated killing by CAR T cells was uniformly higher compared to CAR NK cells in all four target cell lines tested (Fig. 5B–E), analogous to acute stimulation (Fig. 4). After one round of restimulation, CAR T cells also consistently produced much higher amounts of IFN-γ than CAR NK cells, irrespective of the target cell line and almost exclusively through a CAR-mediated effect (Fig. 5F–I).

**Fig. 6:**
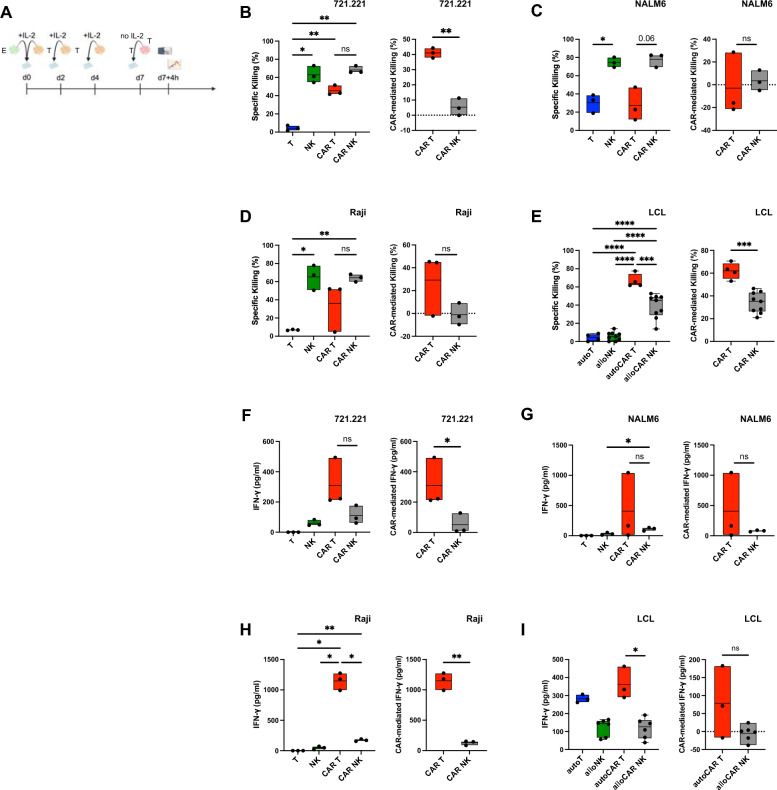
In vitro functionality of CAR T and CAR NK cells after repetitive, long-term restimulation. Expanded cells were repetitively cocultured for one week with the indicated cancer cell lines in the presence of 100 U/ml IL-2. Specific killing after the final 4-h target cell restimulation without IL-2 was assessed by flow cytometry using a membrane-impermeable DNA stain (TO-PRO-3) and PKH26 membrane-labeled target cells. IFN-γ production was measured by ELISA. **A** Schematic of the timeline for multiple restimulations experiments. (B-E) Specific (left) and CAR-mediated (right) killing after repetitive restimulation of 721.221 cells (**B**), NALM6 cells (**C**) or Raji cells (**D**) by anti-CD19 CAR T, CAR NK cells and untransduced controls and of LCL (**E**) by autologous anti-CD19 CAR T cells, allogeneic anti-CD19 CAR NK cells and untransduced controls. (F-I) Overall (left) and CAR-mediated (right) IFN-γ production after final 4-h coculture following repetitive restimulation with 721.221 cells (**F**), NALM6 cells (**G**) or Raji cells (**H**) by anti-CD19 CAR T, CAR NK cells and untransduced controls and with LCL (**I**) by autologous anti-CD19 CAR T cells, allogeneic anti-CD19 CAR NK cells and untransduced controls. Data are from 1 to 2 independent experiments with n = 3–9 donors. Significance by mixed-effects analysis, RM one-way ANOVA with Tukey’s multiple comparisons, ordinary one-way ANOVA with Tukey’s multiple comparisons, two-tailed paired t-test or two-tailed unpaired t-test as appropriate; *p < 0.05, **p < 0.01, ***p < 0.001, ****p < 0.0001. Symbols represent individual donors. Data shown as box and whiskers display the median as a line within the box; whiskers are shown from minimum to maximum data point

To model chronic and recurrent stimulation, effector cells were repetitively exposed to LCL over a period of 7 days (Fig. 6A). Whereas killing of 721.221, NALM6 and Raji cells by untransduced NK and CAR NK cells was sustained in the final rechallenge, CAR T cells displayed decreased killing of NALM6 cells with significantly higher CAR-mediated killing of target cells compared to CAR NK cells against 721.221 cells only (Fig. 6B–D). Nevertheless, CAR-mediated killing of these cancer cell lines by CAR NK cells was virtually absent (Fig. 6B–D). Interestingly, cytotoxicity was maintained at high levels in both autologous CAR T cells and allogeneic CAR NK cells, again with superior killing by CAR T cells (Fig. 6E). While IFN-γ production was reduced across all target cells compared to acute stimulation and single restimulation experiments, CAR T cells continued to produce more in most conditions (Fig. 6F–I). Thus, our data indicated functional exhaustion of CAR T cells in their ability to produce IFN-γ and, to some extent, their capacity for redirected killing, which progressively decreased from single restimulation to repetitive stimulation relative to acute CAR stimulation. IFN-γ production remained mostly unchanged at very low levels in CAR NK cells after chronic CAR stimulation. On the other hand, we did not detect exhaustion in terms of cytotoxicity in neither untransduced NK cells nor CAR NK cells in our in vitro models.

### Anticancer activity of CAR T and CAR NK cells in vivo

**Fig. 7:**
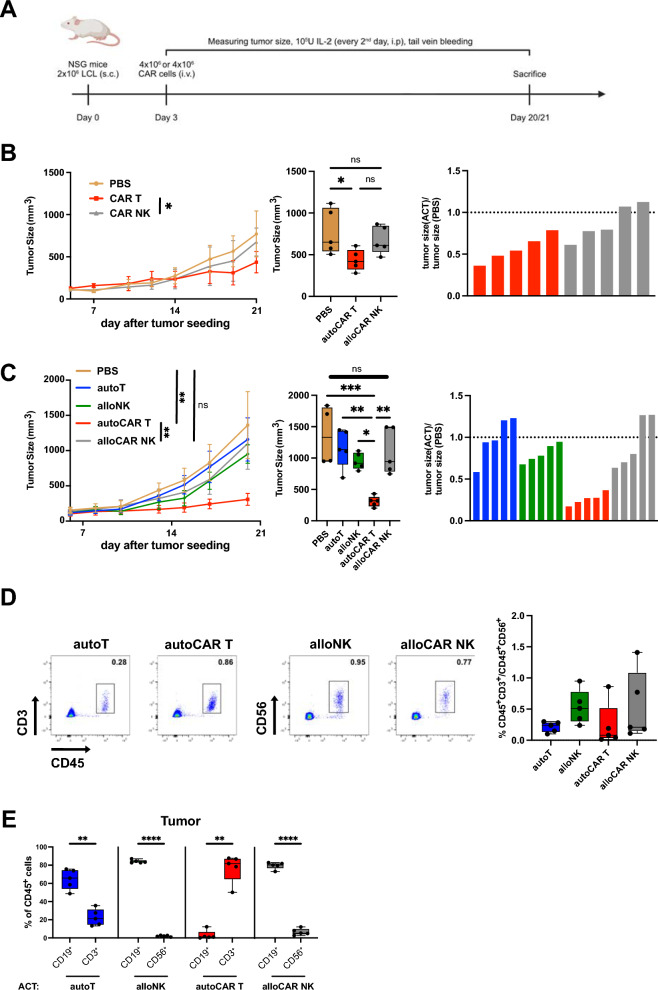
Tumor control in vivo by autologous CAR T cells but not allogeneic CAR NK cells. **A** Schematic outline of in vivo experiments. NSG mice were subcutaneously injected in the flank with 2 × 10^6^ lymphoblastoid tumor cells (in-house generated LCL) and three days after tumor engraftment, mice were treated with ACT of 4 × 10^6^ or 10 × 10^6^ effector cells, being either autologous (T cells products) or allogeneic (NK cell products) to the tumor. Transferred cells were supported by supplementation of IL-2 (1 × 10^5^ IU every second day, i.p.). Tumor growth was monitored with caliper measurements. **B** Tumor growth over time in individual mice treated with 4 × 10^6^ autologous CAR T cells, 4 × 10^6^ allogeneic CAR NK cells or untreated controls (left), tumor size at the endpoint (middle) and tumor size relative to average tumor size in untreated controls (right). **C** Tumor growth over time in individual mice treated with ACT of 10 × 10^6^ effector cells over time (left), tumor size at the endpoint (middle) and tumor size relative to average tumor size in untreated controls (right). **D** Representative dot plots of transferred effector cells identified by CD45 and CD3 or CD56 coexpression (left) and quantification of human cells in peripheral blood at day 14 after tumor engraftment in mice treated with ACT of 10 × 10^6^ effector cells (right). **E** Flow cytometric analysis of tumors showing percentage of CD19^+^ tumor cells, CD3^+^ T cells or CD56^+^ NK cells within the human CD45^+^ compartment in mice receiving ACT of 10 × 10^6^ effector cells as indicated. Data are from 2 independent experiments with n = 15–24 mice (n = 4–5 mice per group). Significance by ordinary one-way ANOVA with Tukey’s multiple comparisons; *p < 0.05, **p < 0.01, ***p < 0.001, ****p < 0.0001. Data shown as box and whiskers display the median as a line within the box; whiskers are shown from minimum to maximum data point

Next, to explore anticancer activity of anti-CD19 autologous CAR T and allogeneic CAR NK cells in vivo, we implanted lymphoblastoid tumor cells (LCL) subcutaneously into NSG mice [35–37], modeling human B cell lymphoma therapy with either autologous CAR T cells [38] or allogeneic CAR NK cells [11]. Mice were treated with adoptive cell transfer (ACT) three days after tumor engraftment and injection of IL-2 to support transferred cells. Transferred effector were either autologous (T cell products) or allogeneic (NK cell products) to the tumor (Fig. 7A). After ACT of 4 × 10^6^ effector cells, we detected autologous CAR T cells in most of the mice at day 10 (Fig. S7A) and all of the mice at day 17 (Fig. S7B) but we were unable to detect allogeneic CAR NK cells at any of these time points. Autologous CAR T cells, but not allogeneic CAR NK cells, reduced tumor growth to a significant but small extent (Fig. 7B and Fig. S8A).

To establish whether increasing the number of transferred autologous CAR T or allogeneic CAR NK cells would increase persistence and tumor control, we adoptively transferred 10 × 10^6^ effector cells. Tumor control was markedly improved in mice treated with autologous CAR T cells, while no effect of allogeneic CAR NK cells was observed, despite the increased number of transferred cells (Fig. 7C and Fig. S8B). Nonetheless, transferred cells could be detected in all of the mice at day 14 after tumor engraftment (Fig. 7D). At the end of experiments, three weeks after tumor engraftment, we harvested blood, spleen and tumors to investigate phenotype of transferred cells in more detail. Autologous CAR T cells showed high levels of the exhaustion markers CD39, PD-1 and Tim-3 in blood, spleen and tumor while control T cells exhibited high expression of these markers only in tumors (Fig. S8C). Activation, as assessed by HLA-DR expression, was higher in CAR T cells than in controls in blood and spleen but similar in tumors. Interestingly, tumor infiltrating CAR T cells had reduced Ki-67 intensity, indicating lower proliferation of CAR T cells relative to controls (Fig. S8D). The percentage of progenitor-like TCF-1^+^ cells was lower in blood in CAR T cells and was further decreased in tumors in both CAR T cells and controls in comparison to blood and spleen (Fig. S8E). Consistent with an effector-like phenotype of tumor infiltrating cells with increased tissue retention, CAR T cells and controls exhibited increased levels of the residency markers CD69 and CD103 (Fig. S8F) and increased levels of the cytolytic effector molecules perforin and granzyme B compared with blood and spleen (Fig. S8G). Overall, these data suggested acquisition of a more activated and exhausted-like T cell state after ACT as compared to the infusion product (Fig. 2D), with exhaustion being more pronounced in CAR T cells across compartments. Transferred NK cells displayed increased CD56 intensity in both CAR NK cells and controls in tumors compared with blood and spleen, indicating activation, while percentage of CD16 and CD57 expression in tumors were decreased (Fig. S9A), thus resembling the infusion product phenotype (Fig. 3D). NKG2A was expressed higher in tumor infiltrating NK cells than in spleen and blood, whereas KIR expression was decreased (Fig. S9B). In line with increased tissue retention, percentages of the residency markers CD69 and CD49a were distinctly higher in tumor infiltrating CAR NK cells and control NK cells (Fig. S9C). Finally, while both transferred NK cell populations displayed high expression Eomes, the transcription factor T-bet was significantly higher relative to controls in tumor infiltrating CAR NK cells, suggesting terminal NK cell maturation [39] (Fig. S9D). Notably, analysis of tumor cell composition indicated eradication of CD19^+^ tumor cells after transfer of CAR T cells, consistent with tumor control as measured by size (Fig. 7C), but not after ACT of any of the other effector cells, including CAR NK cells, as CD19^+^ cells were distinctly depleted only in tumors of mice receiving CAR T cells (Fig. 7E). Together, these data demonstrated efficient tumor control by autologous CAR T cells but not by allogeneic CAR NK cells.

Lastly, we used the NALM6 xenograft model [40] to compare anticancer activity of CAR T cells and CAR NK cells in a systemic leukemia model in vivo. This model is known to be highly responsive to anti-CD19 CAR T cells [41], thus allowing us to use lower numbers of CAR T cells. Because we did not observe tumor control in the LCL lymphoma model with the same number of low- or high-dose CAR NK cells as with CAR T cells, we transferred a higher number of CAR NK cells (10 × 10^6^) compared to CAR T cells (2 × 10^6^) (Fig. S10A). In this setting, tumor control was equal in NALM6-bearing mice transferred with CAR T cells and CAR NK cells, while transfer of neither untransduced T nor untransduced NK cells had an effect, also ruling out prominent intrinsic antitumor activity of NK cells (Fig. S10B, C). Adoptively transferred NK cell products (CAR NK cells and untransduced NK cells) were detected in all mice in peripheral blood and at higher abundance than CAR T cells (Fig. S10D). These findings indicate that CAR NK cells display anticancer activity in vivo but require higher doses than CAR T cells.

## Discussion

Using CAR T and CAR NK cells generated under identical manufacturing processes with a benchmark CAR construct [24] used in an approved CAR T cell product (axi-cel), we demonstrated that CD19 directed human CAR T and CAR NK cells displayed similar cytotoxicity against established CD19^+^ cancer cell lines in vitro in models of both acute and chronic stimulation. However, in modeling a situation of potential future clinical use, allogeneic CAR NK cells exhibited significantly reduced cytotoxicity relative to autologous CAR T cells in vitro against recently established CD19^+^ lymphoblastoid cell lines. The cytotoxic response of CAR NK cells against established CD19^+^ HLA class-I deficient and proficient cancer cell lines might thus be due in relevant part to innate NK cell recognition and not be CAR mediated. Indeed, untransduced NK cells displayed better killing of these cancer cell lines relative to untransduced T cells, hence CAR-mediated cytotoxicity was consistently much higher in CAR T cells than in CAR NK cells. Furthermore, we found that CAR NK cells had a distinctly reduced capacity to produce IFN-γ after coculture with established cancer cell lines as well as in allogeneic settings, but also intrinsically after stimulation with PMA and ionomycin. This is likely to put CAR NK cells at a disadvantage, as the secretion of IFN-γ may be essential for the anticancer activity of CAR cells in regulating adhesion to cancer cells [42], recruitment of endogenous immune cells [43] and antigen spreading [44]. These mechanisms of IFN-γ mediated enhancement of anticancer efficacy or potential loss thereof during adoptive transfer of CAR NK cells mostly rely on interactions with endogenous immune cells, which are difficult to model in vitro or in immunodeficient xenograft models. After chronic repetitive stimulation, CAR T cells secreted less IFN-γ, indicative of exhaustion. However, cytotoxicity was maintained in both autologous CAR T cells and, at reduced level, in allogeneic CAR NK cells. While this is more challenging with CAR T cells, repeated administration of allogeneic CAR NK cells may therefore be reasonable given that they did not exhibit exhaustion with respect to their cytotoxicity. Within this context, we found comparable tumor control by CAR NK cells when using high cell doses as by CAR T cells in a systemic leukemia model. Critically, however, autologous CAR T cells outperformed allogeneic CAR NK cells in our comparative assessment of anticancer activity in vivo in the lymphoma model. We observed reduced persistence of allogeneic CAR NK cells relative to autologous CAR T cells after transfer of lower cell doses, but not after administration of higher cell numbers. Still, these higher cell numbers of CAR NK cells were not sufficient to control tumors in the lymphoma model as did CAR T cells but similarly delayed tumor growth in a systemtic leukemia model.

## Conclusion

In a direct head-to-head comparison of CD19 directed human NK and T cells, we report markedly reduced IFN-γ production, significantly lower CAR-mediated killing by CAR NK cells, and superior anticancer activity of autologous CAR T cells compared to allogeneic CAR NK cells in vivo. Approaches that increase cytotoxicity and IFN-γ production of CAR NK cells might thus be needed to potentiate their anticancer activity.

### Supplementary Information

Supplementary Material 1: Figure S1: (A) Gating strategy for sorting of T and NK cells from PBMCs of healthy donors. (B) Purity of T and NK cells after 3 weeks of expansion and gating for further analysis of T and NK cells. Figure S2: Phenotype by flow cytometry of non-expanded T cells (baseline), expanded untransduced T cells and expanded CAR T cells purified from PBMC after expansion with K562mbIL21 and IL-2 supplementation for 3 weeks. (A) Representative contour plots showing marker expression in expanded untransduced T cells. (B) Bar graphs showing individual marker expression across T cell populations. (C) Bar graph showing individual expression of NK cell markers in T cells at baseline, expanded untransduced human T cells and expanded CAR T cells. Data from 1-3 experiments with n=3-9 donors. Significance by 2way ANOVA with Tukey’s multiple comparisons; *p<0.05, **p<0.01, ***p<0.001, ****p<0.0001. Symbols represent individual donors. Figure S3: (A) Timeline of the generation and expansion of anti-CD19 CAR T cells using CD3/CD28 costimulation. (B) Transduction rates of T cells after CD3/CD28 costimulation. (C) Flow cytometric analysis of CD4 and CD8 expression and percentages of CD4^+^ and CD8^+^ T cells within freshly isolated CD3^+^ T cells (baseline), untransduced T cells and CAR T cells, expanded with CD3/CD28 costimulation (D) Flow cytometric analysis of T cell differentiation and percentages of naïve (T_N_, CD45RA^+^CD62L^+^), central memory (T_CM_, CD45RA^-^CD62L^+^), effector memory (T_EM_, CD45RA^-^CD62L^-^) and T_EMRA_ (CD45RA^+^CD62L^-^) cells within T cell populations before and after expansion with CD3/CD28 costimulation. (E) Bar graphs showing individual marker expression comparing CAR T cells generated with feeder-based expansion (CAR T) or with CD3/CD28 costimulation (CAR T (CD3/CD28_stim_)). (F) CAR T cells were cocultured with the indicated target cell lines at an effector to target ratio of 5:1 for 4 hours. Specific killing was assessed by flow cytometry using a membrane-impermeable DNA stain (TO-PRO-3) and PKH26 membrane-labeled target cells. Specific killing of NALM6 (left) and autologous LCL (right). Data for T cells expanded with K562mbIL21 feeder cells are from 1-3 experiments with n=7-9 donors and data for CD3/CD28 costimulation are from 1 experiment with n=3 donors. Significance by 2way ANOVA with Tukey’s multiple comparisons or two-tailed unpaired t-test as appropriate; *p<0.05, **p<0.01, ***p<0.001, ****p<0.0001. Symbols represent individual donors. Data shown as box and whiskers display the median as a line within the box; whiskers are shown from minimum to maximum data point. Figure S4: (A) Representative contour plots showing marker expression in expanded untransduced NK cells. (B) Bar graphs showing individual marker expression across NK cell populations. Data are from 3 independent experiments with n=5-9 donors. Significance by 2way ANOVA with Tukey’s multiple comparisons; *p<0.05, **p<0.01, ***p<0.001, ****p<0.0001. Symbols represent individual donors. Figure S5: (A) Flow cytometry plots of CD19 expression before (left) and after (right) CRISPR/Cas9-mediated knockout of CD19 in 721.221 cells (B-E) Expanded effector cells were cocultured with the indicated cancer cell lines at the indicated effector to target (E:T) ratios for 4 hours. Specific killing was assessed by flow cytometry using a membrane-impermeable DNA stain (TO-PRO-3) and PKH26 membrane-labeled target cells. Data are from 1 experiment with n=2-3 donors. Significance by 2way ANOVA with Tukey’s multiple comparisons (only CAR T vs. CAR NK is shown); **p<0.01. Figure S6: (A) IFN-γ production of effector cells following stimulation with PMA and ionomycin. (B) Fold expansion of untransduced NK and CAR NK cells after 9 days in either the presence of an IFN-γ blocking antibody (clone B27) or recombinant 10 ng/ml IFN-γ. (C) Expression of IFN-γ-R1 (CD119) on CAR NK cells as assessed by flow cytometry. (D) Specific killing of 721.221 cells (left) and LCL (right) after single restimulation by effector cells in the absence of IL-2. Data are from 1 to 2 independent experiments with n=3-6 donors. Significance by RM one-way ANOVA with Tukey’s multiple comparisons; *p<0.05, **p<0.01, ***p<0.001, ****p<0.0001. Symbols represent individual donors. Data shown as box and whiskers display the median as a line within the box; whiskers are shown from minimum to maximum data point. Figure S7:Tumor-bearing NSG mice were adoptively transferred with 4 x 10^6^ autologous CAR T or 4 x 10^6^ allogeneic CAR NK cells at day 3 after tumor engraftment and presence of transferred cells in peripheral blood was assessed by flow cytometry on day 10 (A) and day 17 (B) after tumor engraftment. 5 mice per group, plots showing data from individual mice. Figure S8: Blood, spleen and tumors were harvested from NSG mice treated with 10 x 10^6^ effector cells (autologous CAR T cells, autologous control T cells, allogeneic CAR NK cells or allogeneic control NK cells) at the end of the experiment and single-cell suspensions analyzed by flow cytometry. (A, B) Tumor growth curves of individual mice treated or not with ACT of 4 x 10^6^ of the indicated effector cells (relates to Figure 7B) (A) and treated or not with ACT of 10 x 10^6^ of the indicated effector cells (relates to Figure 7C) (B). Percentage or mean fluorescence intensity (MFI) of indicated markers within T cells (C-G). 5 mice per group. Significance by two-tailed unpaired t-test; *p<0.05, **p<0.01, ***p<0.001, ****p<0.0001. Data shown as box and whiskers display the median as a line within the box; whiskers are shown from minimum to maximum data point. Figure S9: Blood, spleen and tumors were harvested from NSG mice treated with 10x10^6^ effector cells (autologous CAR T cells, autologous control T cells, allogeneic CAR NK cells or allogeneic control NK cells) at the end of the experiment and single-cell suspensions analyzed by flow cytometry. (A-D) Percentage or mean fluorescence intensity (MFI) of indicated markers. 5 mice per group. Significance by two-tailed unpaired t-test; *p<0.05, **p<0.01, ***p<0.001, ****p<0.0001. Data shown as box and whiskers display the median as a line within the box; whiskers are shown from minimum to maximum data point. Figure S10: (A) Schematic outline of* in vivo *NALM6 experiment. 5 x 10^5^ NALM6-Luc cells were injected intravenously into NSG mice and four days after tumor engraftment, mice were treated with ACT of 2 x 10^6^ (T and CAR T) or 10 x 10^6^ (NK and CAR NK) effector cells from 2 donors each. Transferred cells were supported by supplementation of IL-2 (1 x 10^5^ IU every second day, i.p.). Tumor growth was monitored weekly by IVIS. (B) Bioluminescence measurements of tumor-bearing NSG mice at indicated time points to assess in vivo functionality of adoptively transferred cells. (C) Tumor growth over time measured by IVIS (left) and overall survival (right). (D) Presence of adoptively transferred cells in peripheral blood was assessed by flow cytometry on day 8 after tumor engraftment, plots show individual mice. 4-6 mice per group. Significance by log-rank Mantel–Cox (survival) followed by Bonferroni correction. *p<0.05, **p<0.01.

## Data Availability

All data supporting the findings of this study are available within the paper and its Supplementary Information.
