# Supplementary material for: CAR T cells outperform CAR NK cells in CAR-mediated effector functions in head-to-head comparison
Source: Exp Hematol Oncol. 2024 May 14;13:51. doi: 10.1186/s40164-024-00522-6 (PMC11092129; doi:10.1186/s40164-024-00522-6)
Supplement: Supplementary file 1 — Supplementary Material 1: Figure S1: (A) Gating strategy for sorting of T and NK cells from PBMCs of healthy donors. (B) Purity of T and NK cells after 3 weeks of expansion and gating for further analysis of T and NK cells. Figure S2: Phenotype by flow cytometry of non-expanded T cells (baseline), expanded untransduced T cells and expanded CAR T cells purified from PBMC after expansion with K562mbIL21 and IL-2 supplementation for 3 weeks. (A) Representative contour plots showing marker expression in expanded untransduced T cells. (B) Bar graphs showing individual marker expression across T cell populations. (C) Bar graph showing individual expression of NK cell markers in T cells at baseline, expanded untransduced human T cells and expanded CAR T cells. Data from 1-3 experiments with n=3-9 donors. Significance by 2way ANOVA with Tukey’s multiple comparisons; *p<0.05, **p<0.01, ***p<0.001, ****p<0.0001. Symbols represent individual donors. Figure S3: (A) Timeline of the generation and expansion of anti-CD19 CAR T cells using CD3/CD28 costimulation. (B) Transduction rates of T cells after CD3/CD28 costimulation. (C) Flow cytometric analysis of CD4 and CD8 expression and percentages of CD4+ and CD8+ T cells within freshly isolated CD3+ T cells (baseline), untransduced T cells and CAR T cells, expanded with CD3/CD28 costimulation (D) Flow cytometric analysis of T cell differentiation and percentages of naïve (TN, CD45RA+CD62L+), central memory (TCM, CD45RA-CD62L+), effector memory (TEM, CD45RA-CD62L-) and TEMRA (CD45RA+CD62L-) cells within T cell populations before and after expansion with CD3/CD28 costimulation. (E) Bar graphs showing individual marker expression comparing CAR T cells generated with feeder-based expansion (CAR T) or with CD3/CD28 costimulation (CAR T (CD3/CD28stim)). (F) CAR T cells were cocultured with the indicated target cell lines at an effector to target ratio of 5:1 for 4 hours. Specific killing was assessed by flow cytometry using a membrane-imp [file 40164_2024_522_MOESM1_ESM.pdf]

Supplementary Material

Supplemental Figures 1-10

CAR T cells outperform CAR NK cells in CAR-mediated effector functions in head-to-head comparison

Lukas Egli[1], Meike Kaulfuss[1], Juliane Mietz[1], Arianna Picozzi[1], Els Verhoeyen[2,3], Christian Münz[4], Obinna Chijioke[1,5]

[1]Cellular Immunotherapy, Institute of Experimental Immunology, University of Zürich, Zürich, Switzerland; [2]International Center for Infectiology, research team Enveloped Viruses, Vectors and Innate Responses, Institut national de la santé et de la recherche médicale, Unité 1111, Centre national de la recherche scientifique, Unité mixte de recherche 5308, École Normale Supérieure de Lyon, Université Claude Bernard Lyon 1, University of Lyon, Lyon, France; [3]Université Côte d'Azur, Institut national de la santé et de la recherche médicale, Unité1065, Centre Méditerranéen de Médecine Moléculaire, Nice, France; [4]Viral Immunobiology, Institute of Experimental Immunology, University of Zürich, Zürich, Switzerland; [5]Institute of Medical Genetics and Pathology, University Hospital Basel, Basel, Switzerland

Fig. S1

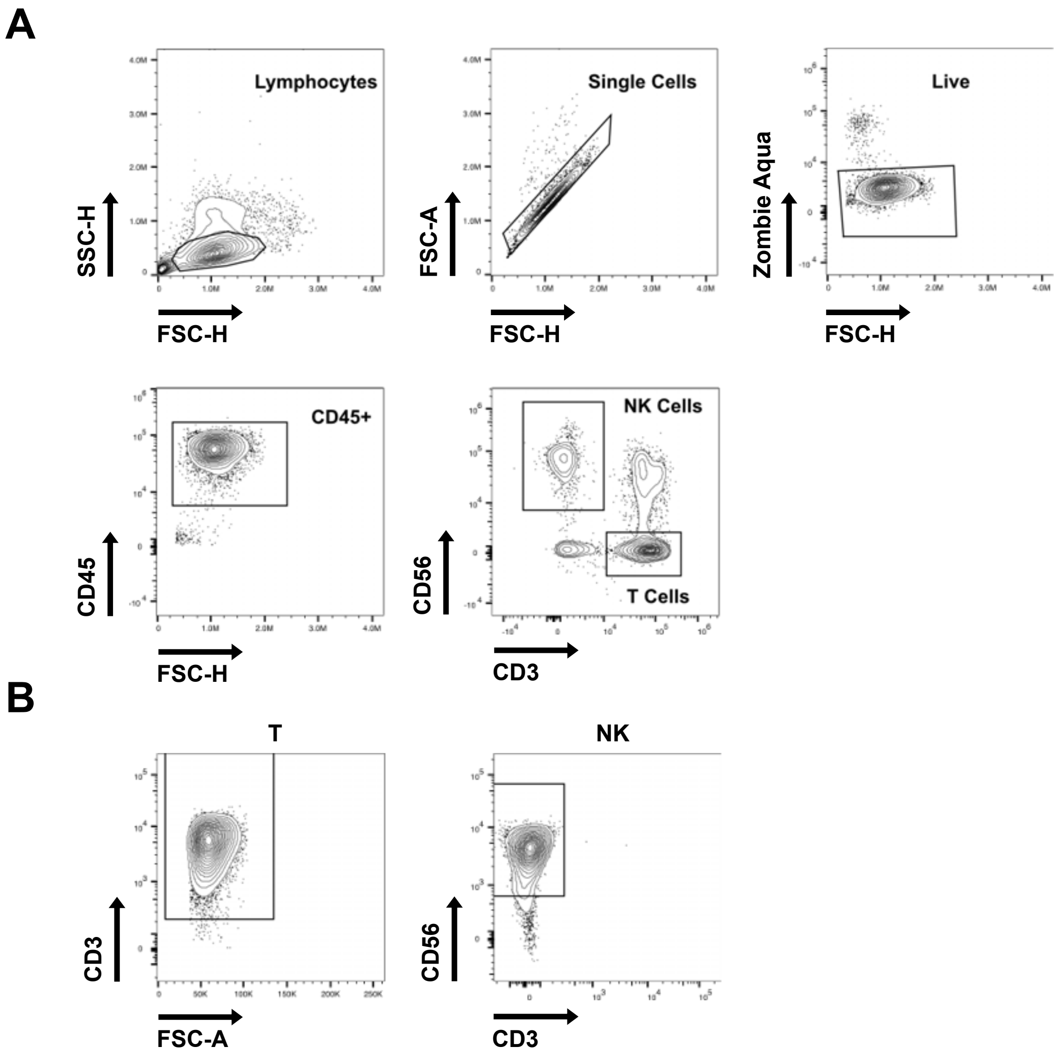
A
Lymphocytes
SSC-H
FSC-H
Single Cells
FSC-A
FSC-H
Live
Zombie Aqua
FSC-H
CD45+
CD45
FSC-H
NK Cells
T Cells
CD56
CD3
B
T
CD3
FSC-A
NK
CD56
CD3

Figure S1:

(**A**) Gating strategy for sorting of T and NK cells from PBMCs of healthy donors. (**B**) Purity of T and NK cells after 3 weeks of expansion and gating for further analysis of T and NK cells.

Fig. S2

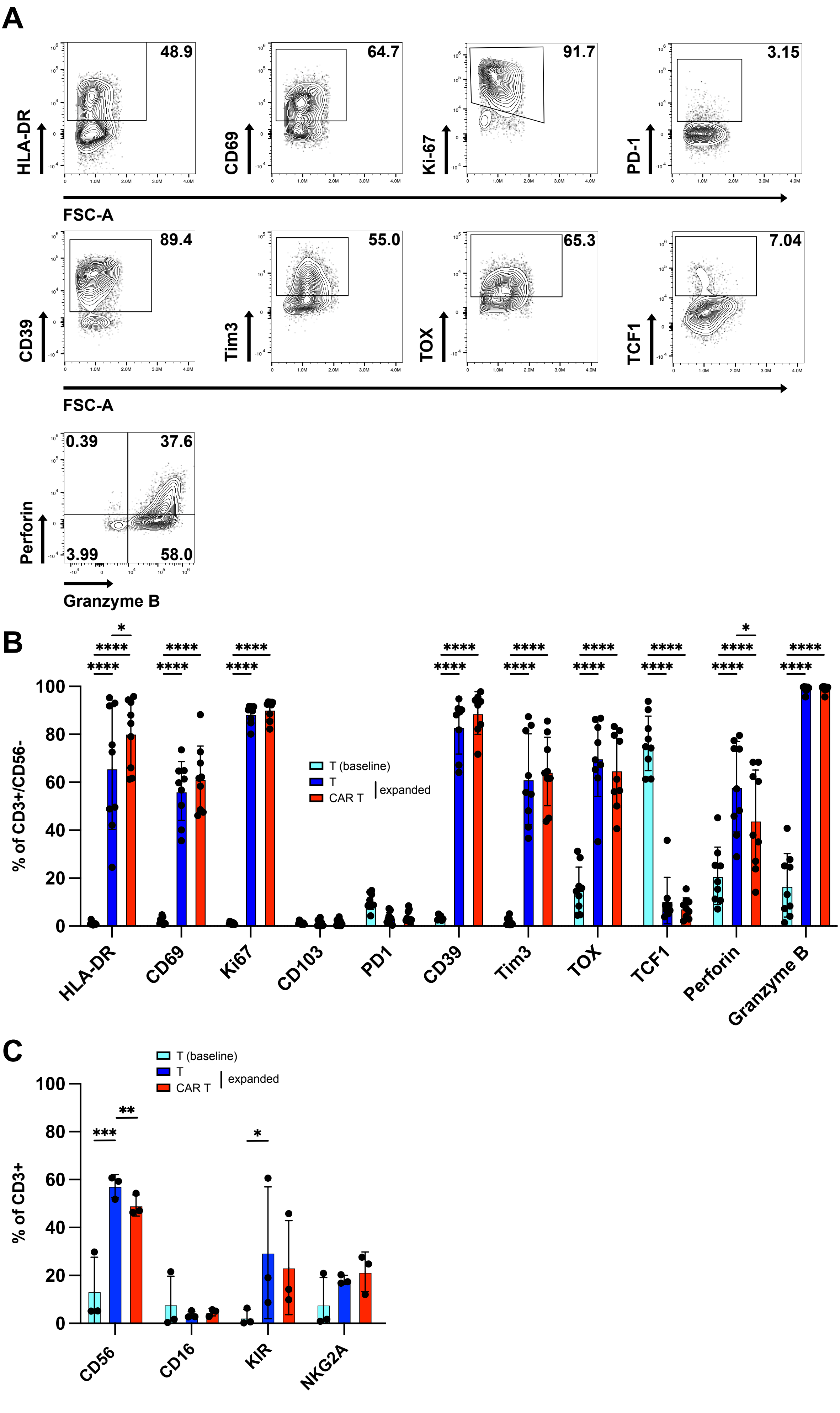

A
HLA-DR
48.9
CD69
64.7
Ki-67
91.7
PD-1
3.15
FSC-A
CD39
89.4
Tim3
55.0
TOX
65.3
TCF1
7.04
FSC-A
Perforin
0.39
37.6
3.99
58.0
Granzyme B
B
% of CD3+/CD56-
T (baseline)
T
CAR T
expanded
HLA-DR
CD69
Ki67
CD103
PD1
CD39
Tim3
TOX
TCF1
Perforin
Granzyme B
C
% of CD3+
T (baseline)
T
CAR T
expanded
CD56
CD16
KIR
NKG2A

Figure S2:

Phenotype by flow cytometry of non-expanded T cells (baseline), expanded untransduced T cells and expanded CAR T cells purified from PBMC after expansion with K562mbIL21 and IL-2 supplementation for 3 weeks. (**A**) Representative contour plots showing marker expression in expanded untransduced T cells. (**B**) Bar graphs showing individual marker expression across T cell populations. (**C**) Bar graph showing individual expression of NK cell markers in T cells at baseline, expanded untransduced human T cells and expanded CAR T cells. Data from 1-3 experiments with n=3-9 donors. Significance by 2way ANOVA with Tukey's multiple comparisons; *$p<0.05$, **$p<0.01$, ***$p<0.001$, ****$p<0.0001$. Symbols represent individual donors.

Fig. S3

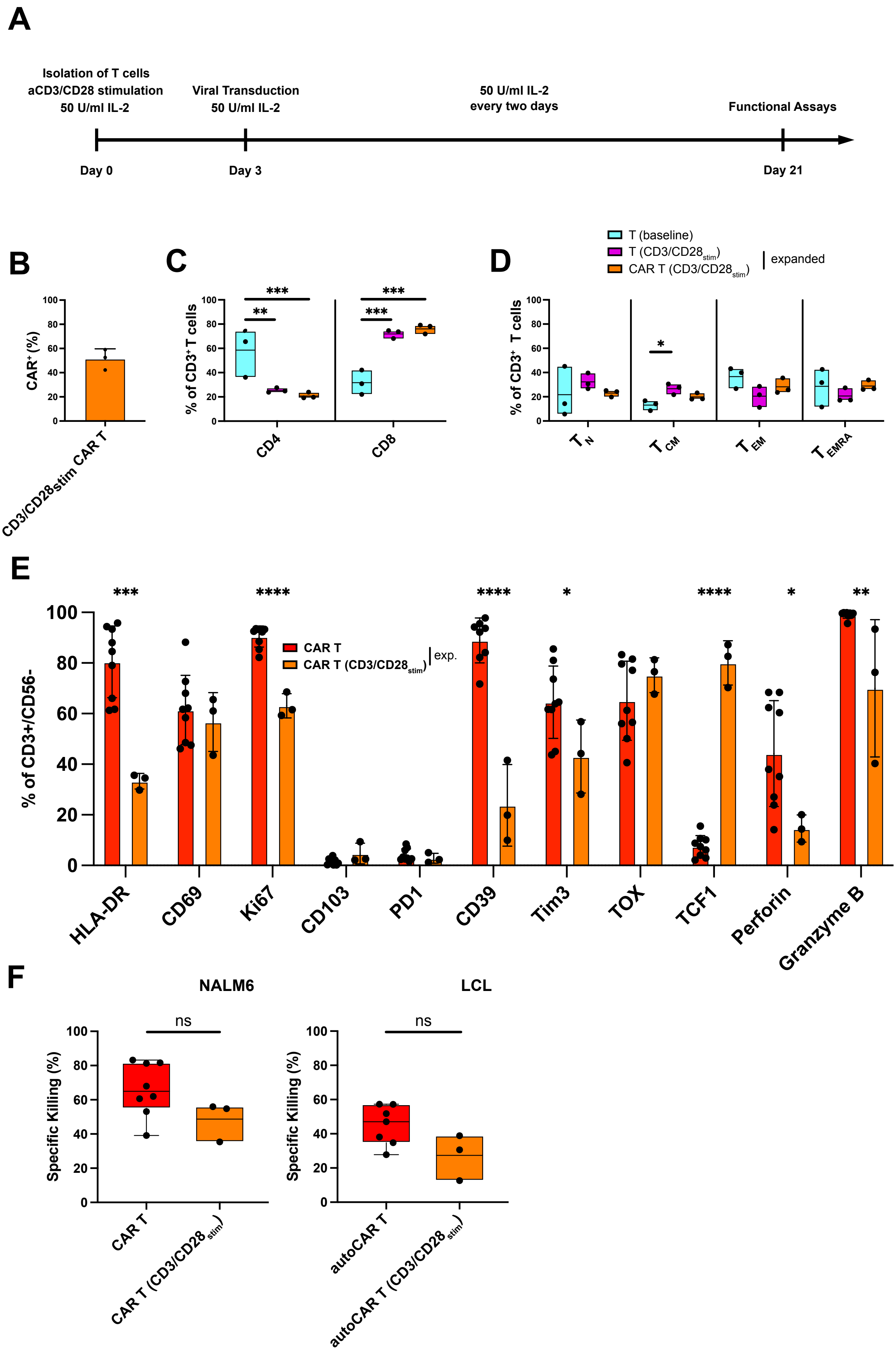
A
Isolation of T cells
aCD3/CD28 stimulation
50 U/ml IL-2
Viral Transduction
50 U/ml IL-2
50 U/ml IL-2
every two days
Functional Assays
Day 0
Day 3
Day 21
B
CAR⁺ (%)
CD3/CD28stim CAR T
C
% of CD3⁺ T cells
CD4
CD8
D
T (baseline)
T (CD3/CD28stim)
CAR T (CD3/CD28stim)
expanded
% of CD3⁺ T cells
T N
T CM
T EM
T EMRA
E
% of CD3+/CD56-
CAR T
CAR T (CD3/CD28stim)
exp.
HLA-DR
CD69
Ki67
CD103
PD1
CD39
Tim3
TOX
TCF1
Perforin
Granzyme B
F
NALM6
LCL
ns
Specific Killing (%)
CAR T
CAR T (CD3/CD28 stim)
autoCAR T
autoCAR T (CD3/CD28 stim)

Figure S3:

(**A**) Timeline of the generation and expansion of anti-CD19 CAR T cells using CD3/CD28 costimulation. (**B**) Transduction rates of T cells after CD3/CD28 costimulation. (**C**) Flow cytometric analysis of CD4 and CD8 expression and percentages of $CD4^{+}$ and $CD8^{+}$ T cells within freshly isolated $CD3^{+}$ T cells (baseline), untransduced T cells and CAR T cells, expanded with CD3/CD28 costimulation (**D**) Flow cytometric analysis of T cell differentiation and percentages of naïve (T_{N}, $CD45RA^{+}CD62L^{+}$), central memory (T_{CM}, $CD45RA^{-}CD62L^{+}$), effector memory (T_{EM}, $CD45RA^{-}CD62L^{-}$) and T_{EMRA} ($CD45RA^{+}CD62L^{-}$) cells within T cell populations before and after expansion with CD3/CD28 costimulation. (**E**) Bar graphs showing individual marker expression comparing CAR T cells generated with feeder-based expansion (CAR T) or with CD3/CD28 costimulation (CAR T ($CD3/CD28_{stim}$)). (**F**) CAR T cells were cocultured with the indicated target cell lines at an effector to target ratio of 5:1 for 4 hours. Specific killing was assessed by flow cytometry using a membrane-impermeable DNA stain (TO-PRO-3) and PKH26 membrane-labeled target cells. Specific killing of NALM6 (left) and autologous LCL (right). Data for T cells expanded with K562mbIL21 feeder cells are from 1-3 experiments with n=7-9 donors and data for CD3/CD28 costimulation are from 1 experiment with n=3 donors. Significance by 2way ANOVA with Tukey's multiple comparisons or two-tailed unpaired t-test as appropriate; *$p<0.05$, **$p<0.01$, ***$p<0.001$, ****$p<0.0001$. Symbols represent individual donors. Data shown as box and whiskers display the median as a line within the box; whiskers are shown from minimum to maximum data point.

Fig. S4

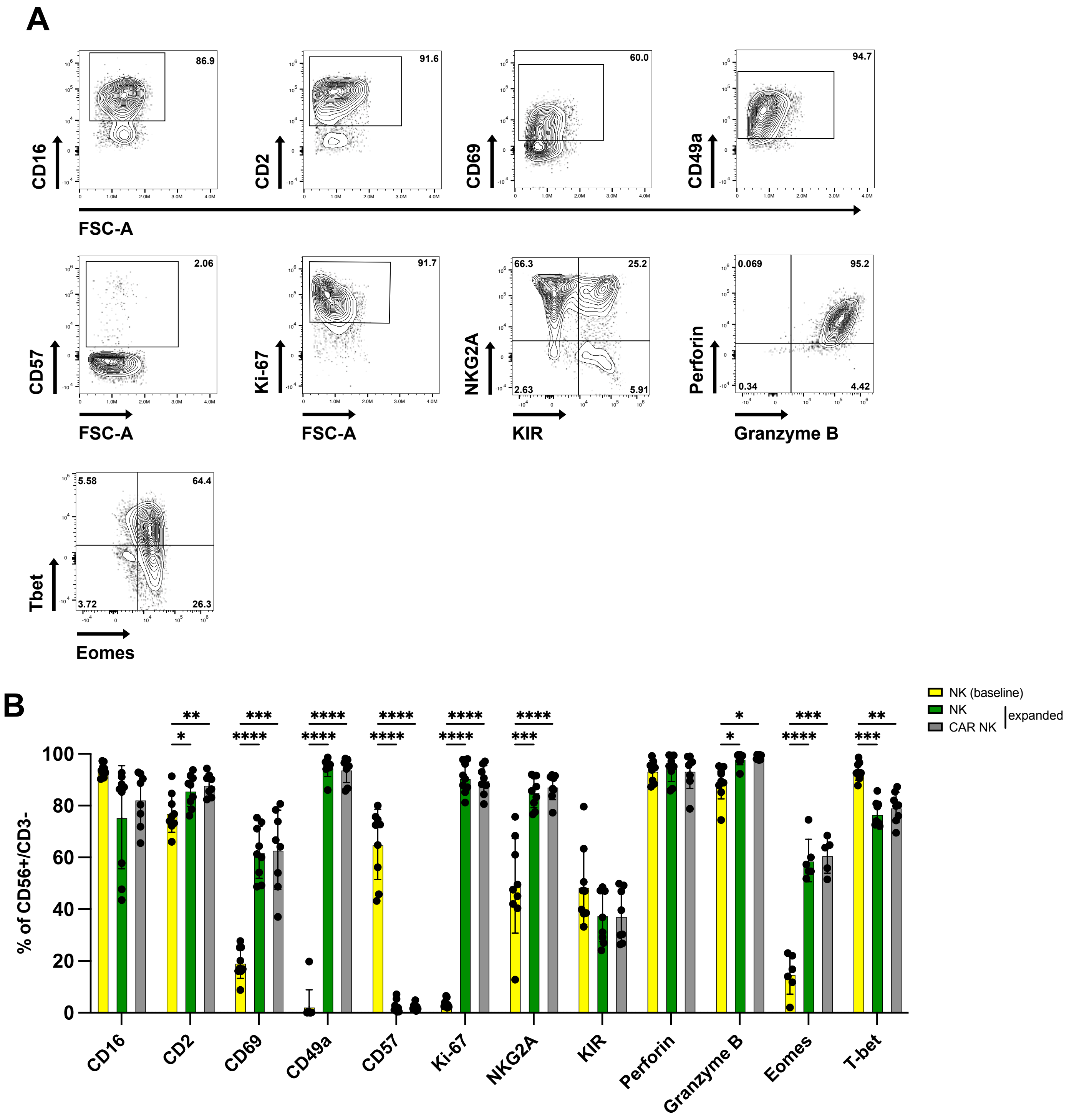
A
86.9
91.6
60.0
94.7
CD16
CD2
CD69
CD49a
FSC-A
2.06
91.7
66.3
25.2
2.63
5.91
0.069
95.2
0.34
4.42
CD57
Ki-67
NKG2A
Perforin
FSC-A
FSC-A
KIR
Granzyme B
5.58
64.4
3.72
26.3
Tbet
Eomes
B
NK (baseline)
NK
CAR NK
expanded
% of CD56+/CD3-
CD16
CD2
CD69
CD49a
CD57
Ki-67
NKG2A
KIR
Perforin
Granzyme B
Eomes
T-bet

Figure S4:

(**A**) Representative contour plots showing marker expression in expanded untransduced NK cells. (**B**) Bar graphs showing individual marker expression across NK cell populations. Data are from 3 independent experiments with n=5-9 donors. Significance by 2way ANOVA with Tukey's multiple comparisons; *p<0.05, **p<0.01, ***p<0.001, ****p<0.0001. Symbols represent individual donors.

Fig. S5

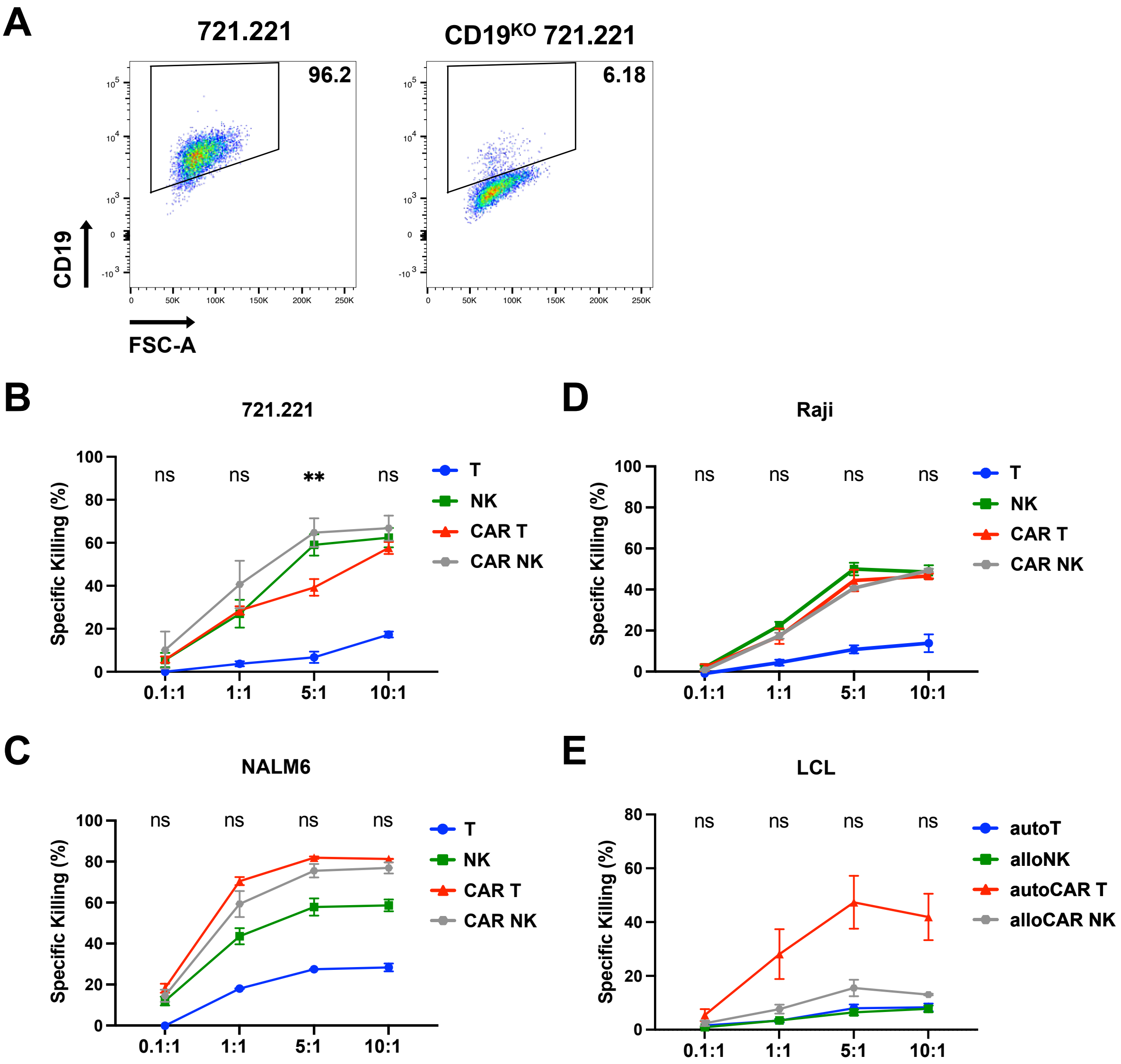
A
721.221
96.2
CD19^{KO} 721.221
6.18
CD19
FSC-A
B
721.221
Specific Killing (%)
ns ns ** ns
0.1:1 1:1 5:1 10:1
T
NK
CAR T
CAR NK
C
NALM6
Specific Killing (%)
ns ns ns ns
0.1:1 1:1 5:1 10:1
T
NK
CAR T
CAR NK
D
Raji
Specific Killing (%)
ns ns ns ns
0.1:1 1:1 5:1 10:1
T
NK
CAR T
CAR NK
E
LCL
Specific Killing (%)
ns ns ns ns
0.1:1 1:1 5:1 10:1
autoT
alloNK
autoCAR T
alloCAR NK

Figure S5:

(**A**) Flow cytometry plots of CD19 expression before (left) and after (right) CRISPR/Cas9-mediated knockout of CD19 in 721.221 cells (**B-E**) Expanded effector cells were cocultured with the indicated cancer cell lines at the indicated effector to target (E:T) ratios for 4 hours. Specific killing was assessed by flow cytometry using a membrane-impermeable DNA stain (TO-PRO-3) and PKH26 membrane-labeled target cells. Data are from 1 experiment with n=2-3 donors. Significance by 2way ANOVA with Tukey's multiple comparisons (only CAR T vs. CAR NK is shown); **$p<0.01$.

Fig. S6

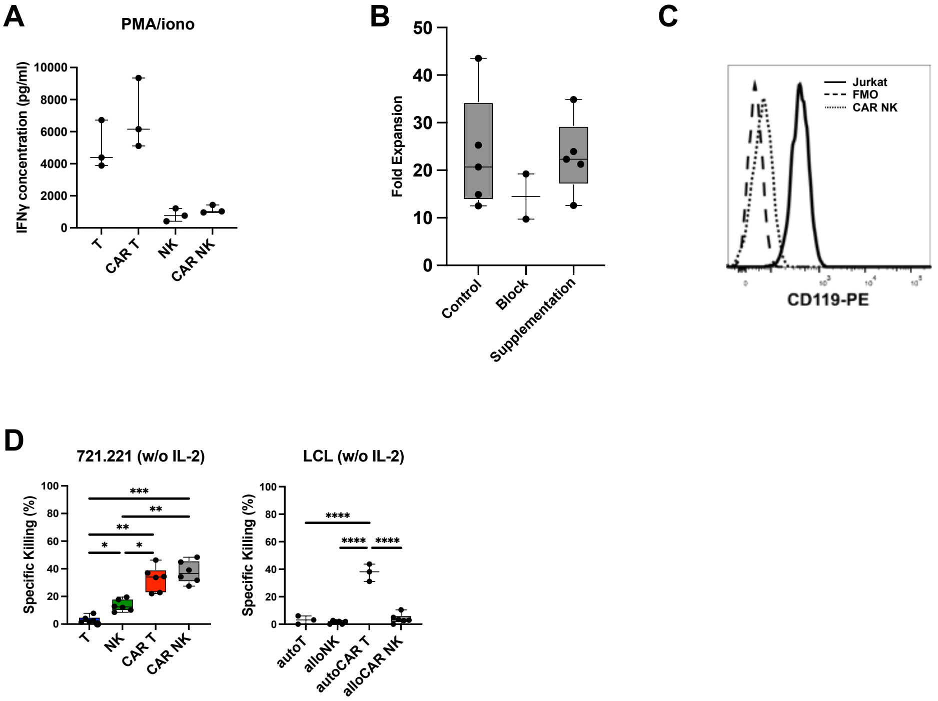

A
PMA/iono
IFNγ concentration (pg/ml)
10000
8000
6000
4000
2000
0
T
CAR T
NK
CAR NK
B
Fold Expansion
50
40
30
20
10
0
Control
Block
Supplementation
C
Jurkat
FMO
CAR NK
CD119-PE
D
721.221 (w/o IL-2)
Specific Killing (%)
100
80
60
40
20
0
***
**
**
*
*
T
NK
CAR T
CAR NK
LCL (w/o IL-2)
****
****
****
autoT
alloNK
autoCAR T
alloCAR NK

Figure S6:

(**A**) IFN-γ production of effector cells following stimulation with PMA and ionomycin. (**B**) Fold expansion of untransduced NK and CAR NK cells after 9 days in either the presence of an IFN-γ blocking antibody (clone B27) or recombinant 10 ng/ml IFN-γ. (**C**) Expression of IFN-γ-R1 (CD119) on CAR NK cells as assessed by flow cytometry. (**D**) Specific killing of 721.221 cells (left) and LCL (right) after single restimulation by effector cells in the absence of IL-2. Data are from 1 to 2 independent experiments with n=3-6 donors. Significance by RM one-way ANOVA with Tukey's multiple comparisons; *$p<0.05$, **$p<0.01$, ***$p<0.001$, ****$p<0.0001$. Symbols represent individual donors. Data shown as box and whiskers display the median as a line within the box; whiskers are shown from minimum to maximum data point.

Fig. S7

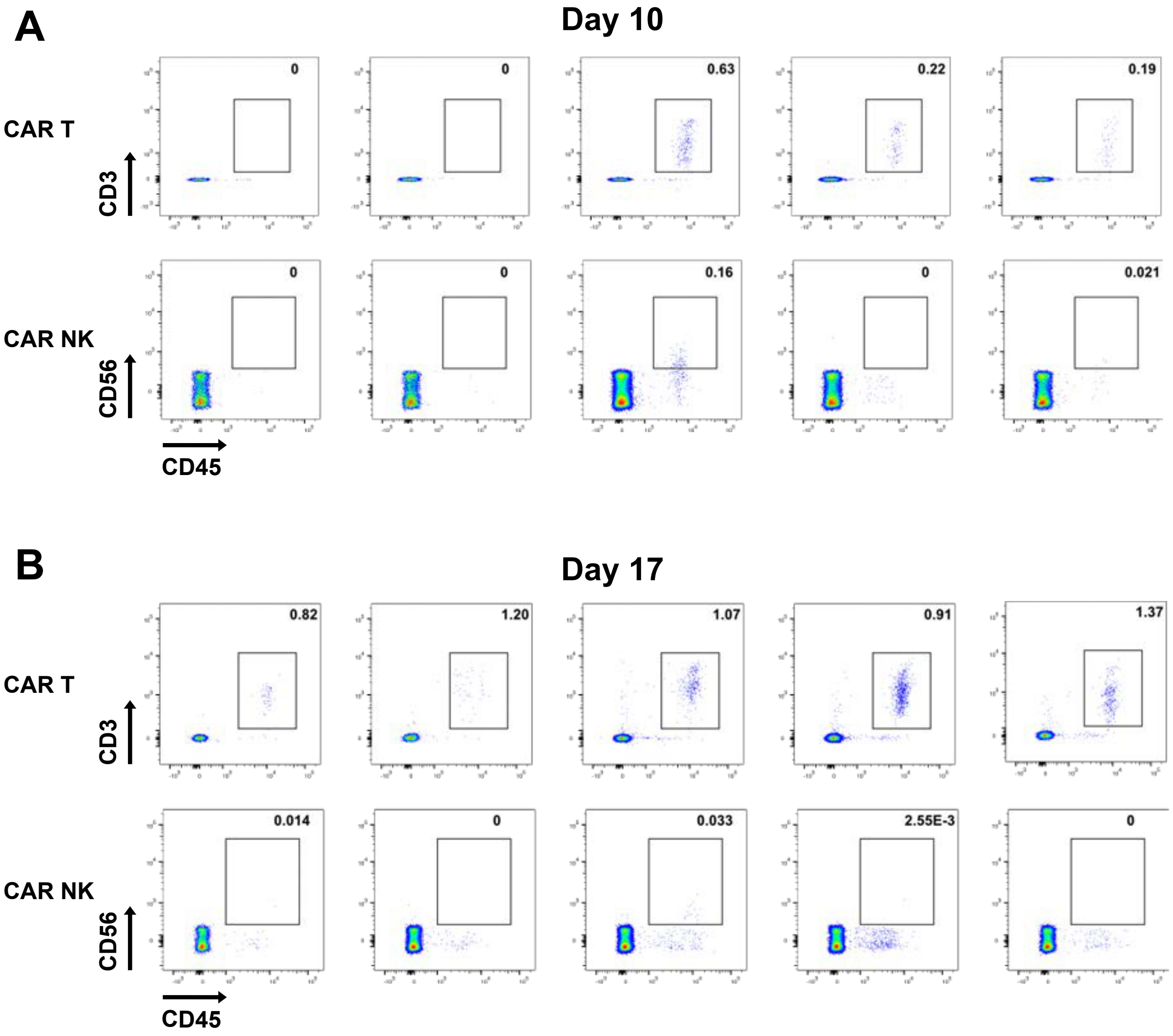
A
Day 10
CAR T
CD3
0
0
0.63
0.22
0.19
CAR NK
CD56
0
0
0.16
0
0.021
CD45
B
Day 17
CAR T
CD3
0.82
1.20
1.07
0.91
1.37
CAR NK
CD56
0.014
0
0.033
2.55E-3
0
CD45

Figure S7:

Tumor-bearing NSG mice were adoptively transferred with 4 x 10^6 autologous CAR T or 4 x 10^6 allogeneic CAR NK cells at day 3 after tumor engraftment and presence of transferred cells in peripheral blood was assessed by flow cytometry on day 10 (**A**) and day 17 (**B**) after tumor engraftment. 5 mice per group, plots showing data from individual mice.

Fig. S8

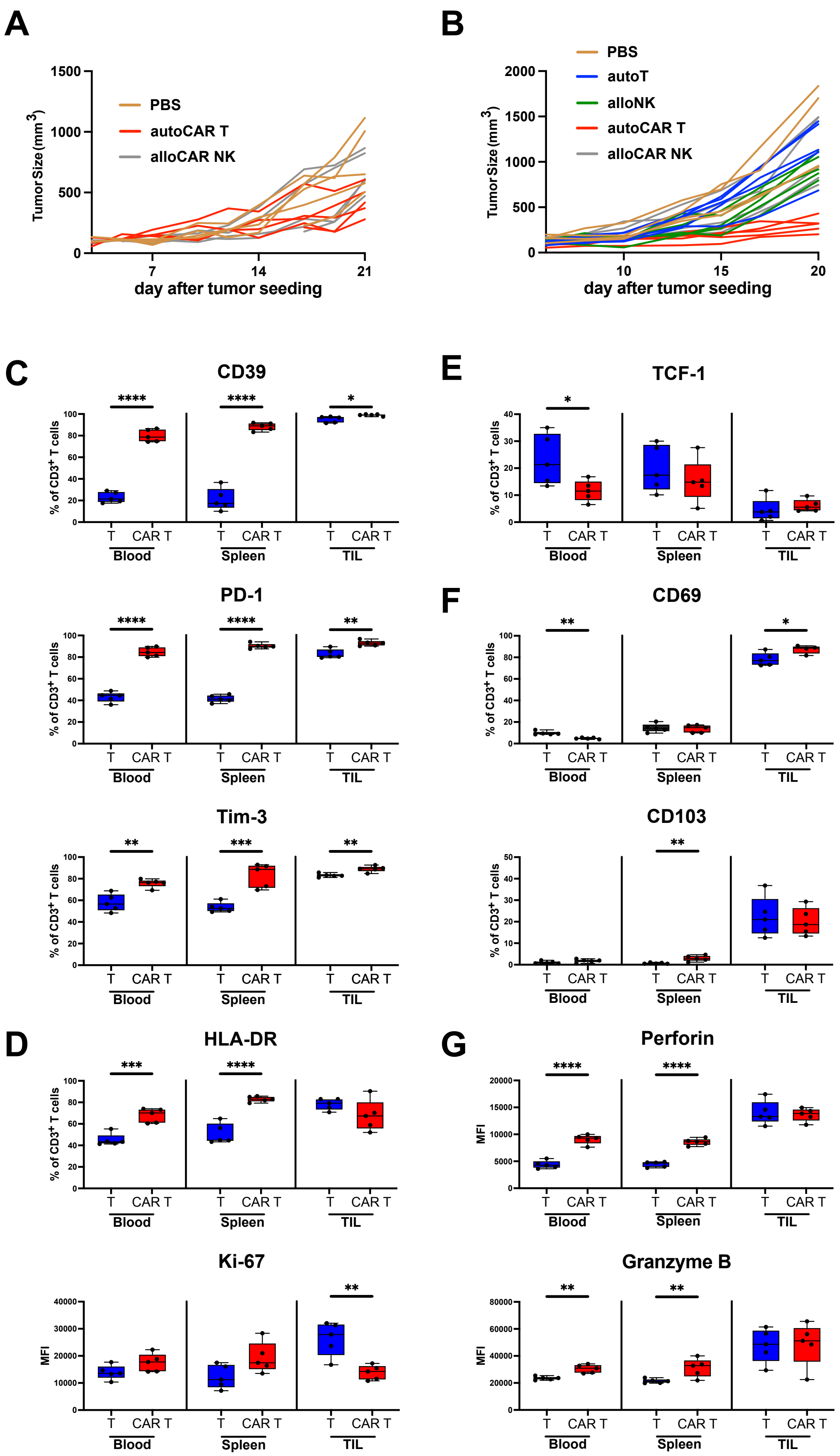

A
Tumor Size (mm^3)
1500
1000
500
0
7
14
21
day after tumor seeding
PBS
autoCAR T
alloCAR NK
B
Tumor Size (mm^3)
2000
1500
1000
500
0
10
15
20
day after tumor seeding
PBS
autoT
alloNK
autoCAR T
alloCAR NK
C
CD39
PD-1
Tim-3
D
HLA-DR
Ki-67
E
TCF-1
F
CD69
CD103
G
Perforin
Granzyme B
% of CD3$^+$ T cells
MFI
T
CAR T
Blood
Spleen
TIL
****
***
**
*

Figure S8:

Blood, spleen and tumors were harvested from NSG mice treated with 10 x 10^6 effector cells (autologous CAR T cells, autologous control T cells, allogeneic CAR NK cells or allogeneic control NK cells) at the end of the experiment and single-cell suspensions analyzed by flow cytometry. (A, B) Tumor growth curves of individual mice treated or not with ACT of 4 x 10^6 of the indicated effector cells (relates to Figure 7B) (**A**) and treated or not with ACT of 10 x 10^6 of the indicated effector cells (relates to Figure 7C) (**B**). Percentage or mean fluorescence intensity (MFI) of indicated markers within T cells (**C-G**). 5 mice per group. Significance by two-tailed unpaired t-test; *$p<0.05$, **$p<0.01$, ***$p<0.001$, ****$p<0.0001$. Data shown as box and whiskers display the median as a line within the box; whiskers are shown from minimum to maximum data point.

Fig. S9

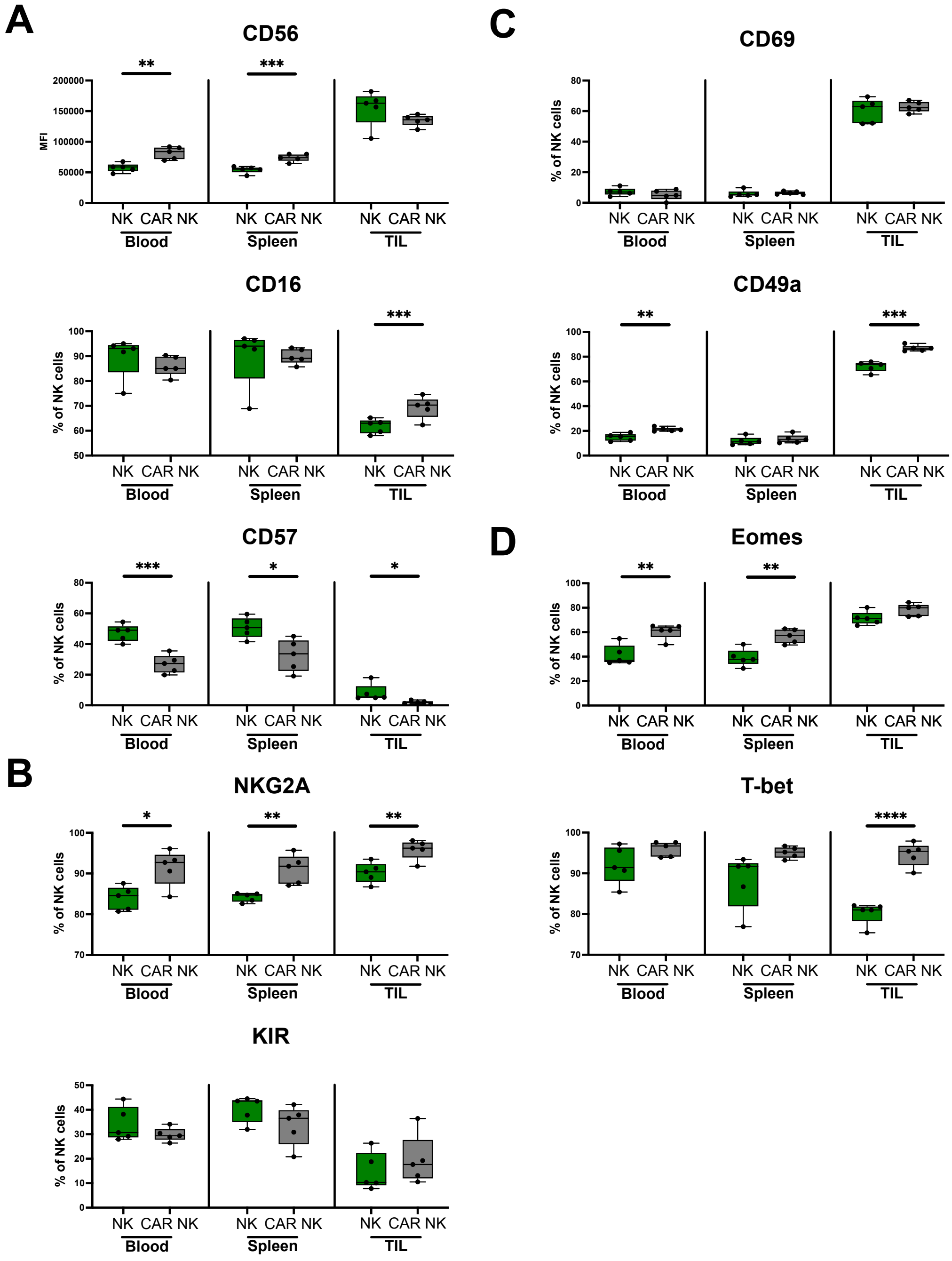
A
C
D
B
CD56
CD69
CD16
CD49a
CD57
Eomes
NKG2A
T-bet
KIR
MFI
% of NK cells
NK CAR NK
Blood
Spleen
TIL
**
***
*
****

Figure S9:

Blood, spleen and tumors were harvested from NSG mice treated with $10x10^6$ effector cells (autologous CAR T cells, autologous control T cells, allogeneic CAR NK cells or allogeneic control NK cells) at the end of the experiment and single-cell suspensions analyzed by flow cytometry. (A-D) Percentage or mean fluorescence intensity (MFI) of indicated markers. 5 mice per group. Significance by two-tailed unpaired t-test; *$p<0.05$, **$p<0.01$, ***$p<0.001$, ****$p<0.0001$. Data shown as box and whiskers display the median as a line within the box; whiskers are shown from minimum to maximum data point.

Fig. S10

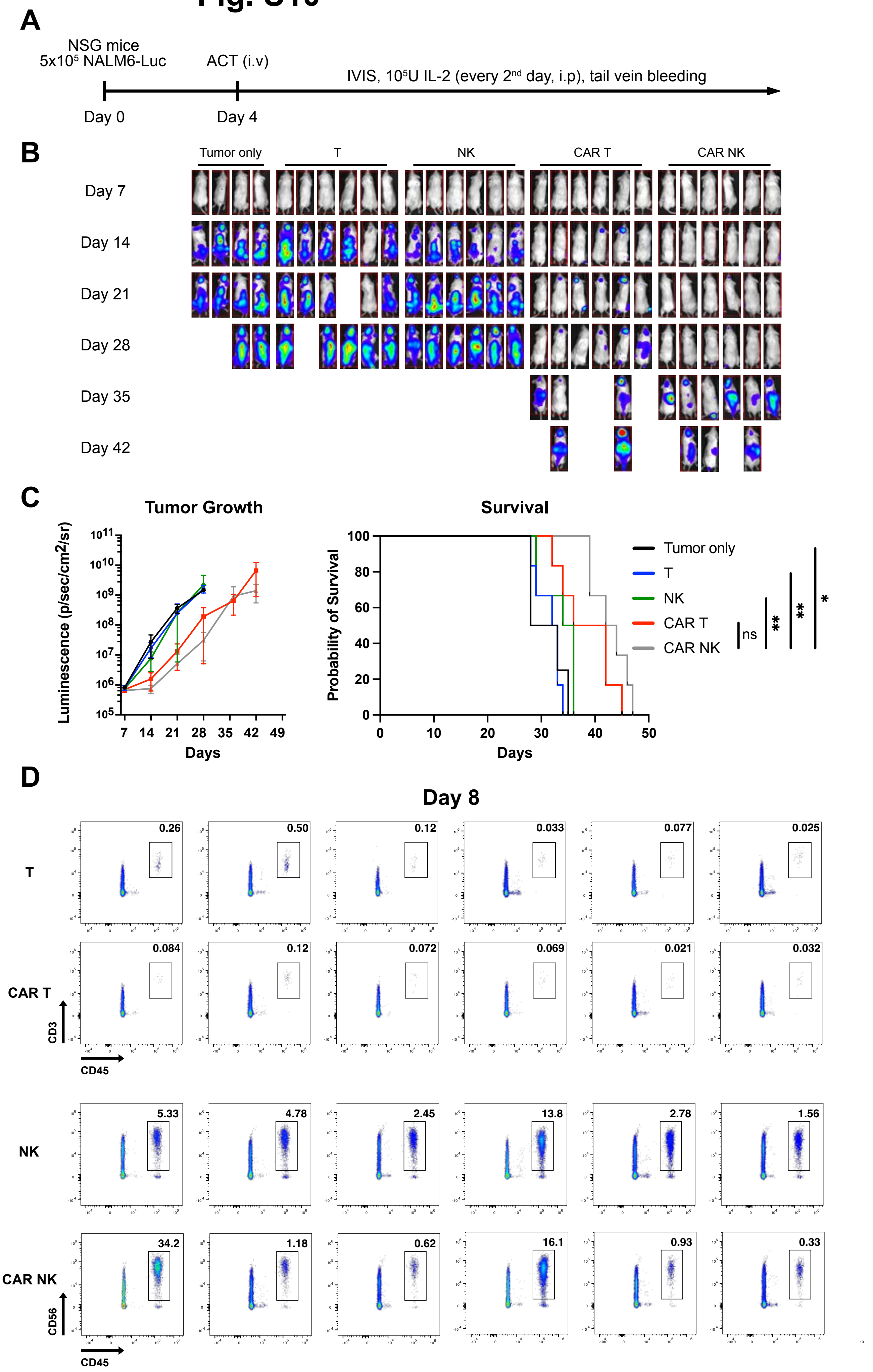
A
NSG mice
5x10⁵ NALM6-Luc
ACT (i.v)
IVIS, 10⁵U IL-2 (every 2nd day, i.p), tail vein bleeding
Day 0
Day 4
B
Tumor only
T
NK
CAR T
CAR NK
Day 7
Day 14
Day 21
Day 28
Day 35
Day 42
C
Tumor Growth
Luminescence (p/sec/cm2/sr)
Days
Survival
Probability of Survival
Days
Tumor only
T
NK
CAR T
CAR NK
ns
**
**
*
D
Day 8
T
CAR T
NK
CAR NK
CD3
CD45
CD56
CD45
0.26
0.50
0.12
0.033
0.077
0.025
0.084
0.12
0.072
0.069
0.021
0.032
5.33
4.78
2.45
13.8
2.78
1.56
34.2
1.18
0.62
16.1
0.93
0.33

Figure S10:

(**A**) Schematic outline of *in vivo* NALM6 experiment. 5 x 10^5 NALM6-Luc cells were injected intravenously into NSG mice and four days after tumor engraftment, mice were treated with ACT of 2 x 10^6 (T and CAR T) or 10 x 10^6 (NK and CAR NK) effector cells from 2 donors each. Transferred cells were supported by supplementation of IL-2 (1 x 10^5 IU every second day, i.p.). Tumor growth was monitored weekly by IVIS. (**B**) Bioluminescence measurements of tumor-bearing NSG mice at indicated time points to assess in vivo functionality of adoptively transferred cells. (**C**) Tumor growth over time measured by IVIS (left) and overall survival (right). (**D**) Presence of adoptively transferred cells in peripheral blood was assessed by flow cytometry on day 8 after tumor engraftment, plots show individual mice. 4-6 mice per group. Significance by log-rank Mantel–Cox (survival) followed by Bonferroni correction. *p<0.05, **p<0.01.
